# Etoposide-induced protein 2.4 homolog promotes argininosuccinate synthase 1 and cancer cell survival upon arginine deprivation

**DOI:** 10.1186/s11658-025-00726-6

**Published:** 2025-04-19

**Authors:** Vu T. A. Vo, Le Nhat Tran, Thu Thanh Bui, Han-Woong Lee, Yangsik Jeong

**Affiliations:** 1 Department of Biochemistry, Wonju College of Medicine, Yonsei University, Wonju, Republic of Korea; 2 Department of Global Medical Science, Wonju College of Medicine, Yonsei University, Wonju, Republic of Korea; 3 Organelle Medicine Research Center, Wonju College of Medicine, Yonsei University, Wonju, Republic of Korea; 4 Department of Biochemistry, College of Life Science and Biotechnology, Yonsei University, Seoul, Republic of Korea; 5 Institute of Mitochondrial Medicine, Wonju College of Medicine, Yonsei University, Wonju, Republic of Korea; 6 ONCOin, Ltd., Startup cube #2 - 204, 1 Kangwondaehakgil, Chuncheon, Republic of Korea

**Keywords:** EI24, ASS1, Arginine, Cancer metabolism

## Abstract

**Background:**

Arginine auxotrophy has been reported in a subset of cancers with inherently defective de novo arginine synthesis. However, the use of arginine deprivation therapy seems to be unequally effective, partially owing to the resistance acquired by cancer cells. Study of underlying factors involved in this response thus becomes of utmost importance. Meanwhile, the function of etoposide-induced 2.4 homolog (EI24) in cancer metabolism, and specifically in arginine metabolism, remains unknown.

**Methods:**

EI24 was overexpressed in cancer cells using a doxycycline-inducible system or adenovirus transduction, while siRNA was used to knockdown EI24. Amino acid(s) deprivation medium was exploited with a cell viability assay to check the reliance of cancer cell survival on arginine. Protein expression and activation were examined through western blot and co-immunoprecipitation blot. Furthermore, global and specific protein translation were assessed through the SUnSET assay and polysome fractionation analysis. Gene expression and arginine level were downloaded from public cancer datasets for in silico validation including gene set enrichment and survival analysis to objectively evaluate the association between EI24 and arginine metabolism.

**Results:**

EI24 promoted cancer survival under arginine starvation. Mechanistically, EI24 replenished translation of argininosuccinate synthase 1 (ASS1) by inducing the inactive *S*-nitrosylated form of phosphatase and tensin homolog (PTEN), leading to release of the phosphoinositide 3-kinase (PI3K)/protein kinase B (AKT) axis. This tumor-promoting action of EI24 could be found in multiple ASS1-deficient cancer cells regardless of p53 status. Furthermore, expression of EI24 was linked to enrichment of arginine metabolism pathway as well as poor survival of patients with cancer across various cancer types, suggesting its role in cancer resistance to arginine deprivation.

**Conclusions:**

This study is the first to report the role of EI24 in promoting cancer survival via translational regulation of the metabolic enzyme ASS1, thus paving a route for further investigation into the link between EI24 and cancer metabolism.

**Graphical Abstract:**

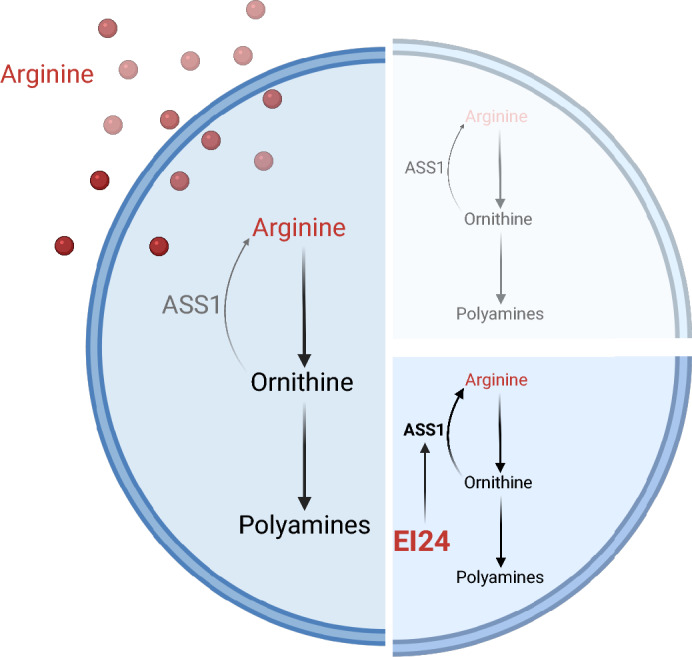

**Supplementary Information:**

The online version contains supplementary material available at 10.1186/s11658-025-00726-6.

## Background

Cancer cells have been widely recognized for their inherent rewiring mechanism to utilize nutrients by robust and complex pathways [1, 2], which permits cancer growth and dissemination under constrained metabolic conditions in the microenvironment [3]. While the phenomenal observation of Otto Warburg and colleagues that tumor cells abnormally increased glucose uptake toward lactate production [4] placed glucose in the spotlight regarding cancer metabolism, numerous studies have also revealed remarkable changes in the metabolism of lipids and amino acid(s) [5, 6] and recently of semi-essential arginine [7, 8]. The early notions that arginine promoted tumorigenesis in patients [9] and that arginine preferably moved from blood to cancerous tissues [10] but not normal counterparts [11] have long implied the immense potential of this amino acid in tumor pathology. In parallel with its well-known function in the urea cycle, as one of the intermediates carrying ammonia to form urea [12], arginine has also been reported to serve as a precursor for polyamine and nitric oxide, both being permissive for cancer invasion [13–15]. Thus, starving cells of arginine has been considered to be an appealing regime that is currently under clinical investigation for various types of cancer [16–19], including a successful phase 3 trial in mesothelioma [20]. Strikingly, tumors have been reported to acquire resistance to such treatment, although the underlying molecular mechanisms remained largely unclear [21].

Cancer cells become reliant on arginine when their excessive demand coincides with the impairment of intrinsic arginine production controlled by the rate-limiting enzyme argininosuccinate synthase 1 (ASS1) [13]. As its name suggested, intact ASS1 incorporates aspartate to citrulline to form argininosuccinate, the immediate precursor of arginine. This process supposedly nourishes cells with de novo synthesized arginine [12]. Of note, tumor cells evolved to favor the shunting of aspartate to nucleotide instead of arginine synthesis [22] following silencing of ASS1 [23–25]. Paradoxically, upon arginine deprivation, resurgence of ASS1 has been reported and considered a direct mechanism of acquired resistance of cancer to therapy [26]. While transcription of ASS1 has been well established as occurring through several factors such as p53 [27, 28], c-MYC [29–31], or hypoxia-inducible factor 1-alpha (Hif1α) [32], evidence on translational regulation of ASS1 is alarmingly missing.

Herein, we report the enhanced regulation of ASS1 protein translation in cancer cells upon arginine starvation, interestingly via a novel function of etoposide-induced protein 2.4 homolog (EI24). Identified as a target of p53 [33], EI24 was initially known for its involvement in apoptosis [34] and autophagy [35]. More recently, this protein was reported to tightly modulate the contact sites of endoplasmic reticulum (ER) and mitochondria, thus contributing to calcium homeostasis and cell adaptation to ER stress [36–38]. Of note, while EI24 has been controversially studied for being a tumor suppressor [39, 40] or promoter [41, 42], its function in metabolic reprogramming is not known elsewhere. Hence, by showing that EI24 may significantly benefit cancer cell adaptation to restricted arginine levels, this study hopes to shed the first light on the role of EI24 in the context of cancer metabolism.

## Materials and methods

### Cell lines and reagents

Breast cancer cells ZR-75-1 were from Professor Jaewhan Song (Yonsei University). MCF7, MDA-MB-231, BT-549, Hs 578T, and lung cancer cells A549, H1299, and H1975 were kindly provided by Professor John Minna (University of Texas Southwestern Medical Center). Renal cell carcinoma cells ACHN, Caki-1, and Caki-2 were obtained from the Korean Cell Line Bank (KCLB number 21611, 30046, and 30047). MDA-MB-231 cells with doxycycline-inducible EI24 were generated by cloning human EI24 cDNA to form the pCW57-RFP-P2A-EI24-EGFP vector followed by lentivirus-mediated induction of a stable cell line. All the cells were cultured at 37 °C with 5% CO_2_ in Roswell Park Memorial Institute (RPMI) medium with 10% fetal bovine serum (FBS) for H1975 and in Dulbecco’s modified Eagle’s medium (DMEM) with 5% FBS for other cell lines. Lysine (L8662), spermidine (S2501), cycloheximide (CHX, C7698), MG-132 (M7449), and cobalt(II) chloride (C8661) were purchased from Sigma. Difluoromethylornithine (DFMO, 16889) and DETA NONOate (82120) were from Cayman. *N*-nitro-l-arginine methylester (L-NAME, sc-200333) and deferoxamine (sc-203331) were from Santa Cruz. Bafilomycin A1 (Baf, ab120497) was from Abcam.

### Adenovirus, plasmids, and transfection

TOPO™ cloning was performed to produce EI24-expressing pENTR™/SD/D-TOPO™ vector prior to LR reaction to insert EI24-expressing sequence into pAd/CMV/V5. pAd/CMV/V5-EI24 was then digested by *Pac*I and transfected to HEK293-A to produce EI24-expressing adenovirus. Constructs of hotspot mutation of p53 were generated using the site-directed mutagenesis method described previously [43] with the template construct pcDNA-flag-p53, a gift from Professor John Minna (University of Texas Southwestern Medical Center), using EzPCR™ HF 5× Master Mix (Elpis). X-tremeGENE ™ HP DNA transfection reagent (Roche) was mixed with plasmids, followed by transfection to the cells in culture media without FBS and antibiotics.

### Knockdown of EI24 using siRNA

Cells were plated 1 day prior to reverse transfection with 30 nM of either control siRNA or EI24 siRNA (Bioneer) in culture media lacking FBS and antibiotics. siRNA was delivered to the cells using X-tremeGENE™ siRNA transfection reagent (Roche). After 6 h, the medium was replaced with complete media, and the cells were used for treatment 48 h post-transfection.

### Amino acid deprivation medium preparation

Amino acid-free DMEM or RPMI powder (D9800-27 and R9010-01, USBiological) was reconstituted and supplemented with glucose and sodium bicarbonate according to the manufacturer’s instructions. Each individual amino acid (Sigma) was prepared as a 100× stock solution in water and then added to reach the desired final concentration of DMEM or RPMI. Deprived amino acid was omitted from the media.

### Quantitative polymerase chain reaction (qPCR)

TRIzol reagent was used to extract RNA from the cells after treatment. RNA was then used as template for cDNA synthesis using ReverTra Ace™ qPCR RT Master Mix with gDNA Remover (Toyobo). qPCR was conducted on a QuantStudio 6 Flex system with TOPreal™ SYBR Green qPCR High-ROX PreMIX (Enzynomics) added to triplicates of reaction. Fold change of gene expression was calculated by the ΔΔCt method with *18S* serving as internal control.

Sequences of specific primers for each gene were as follows:

*18S*: forward 5′-ACCGCAGCTAGGAATGGA- 3′; reverse 5′-GCCTCAGTTCCGAAAACC- 3′.

*EI24*: forward 5′-AATGCACCAGCGGTTGTCTAA- 3′; reverse 5′-GATAGAGAAAAGGCAGCCACTGA- 3′.

*ARG2*: forward 5′-ATAGGAGCCCCGTTCTCACA- 3′; reverse 5′-CTTCTCTTATGGCAGCGGGA- 3′.

*ASL*: forward 5′-GACCATCAGCCCCCTGTTC- 3′; reverse 5′-GGCACCATACTGCTCCACACT- 3′.

*ASS1*: forward 5′-GGAACGATCAGGTCCGGTTT- 3′; reverse 5′-CGTGTTGCTTTGCGTACTCC- 3′.

*CPS1*: forward 5′-TGGCAGCATTGACCTAGTGA- 3′; reverse 5′-TGCACAGCTTCAGCAAAAAG- 3′.

*ODC1*: forward 5′-ATGCCCGCTGTGTTTTTGAC- 3′; reverse 5′-TACGCCGGTGATCTCTTCAA- 3′.

*OTC*: forward 5′-AGCGCATGGAGGCAATGTAT- 3′; reverse 5′-GGCAGCAACTTTAGCAGTCTTC- 3′.

*OAT*: forward 5′-GGATGCTTGGAAGGTGTGTC- 3′; reverse 5′-TGGACTCTCGAAGCTCATCC- 3′.

*ACTB*: forward 5′-GGCATCCTCACCCTGAAGTA- 3′; reverse 5′-GGGGTGTTGAAGGTCTCAAA- 3′.

*VEGFA*: forward 5′-CTTGCCTTGCTGCTCTACCT- 3′, reverse 5′-TCCATGAACTTCACCACTTCGT- 3′.

### Western blot

Cells were lysed in RIPA lysis buffer (150 mM NaCl, 1% Triton X- 100, 0.5% sodium deoxycholate, 0.1% sodium dodecyl sulfate (SDS), 50 mM Tris pH 8) supplementing with protease inhibitors (Sigma) and the phosSTOP (Roche) phosphatase inhibitor cocktail. Protein concentrations were determined using the Pierce™ bicinchoninic acid (BCA) protein assay kit (Thermo) before proceeding to western blot. The nitrocellulose membranes with proteins transferred from SDS gels were blocked in 5% skim milk before primary and secondary antibodies incubation. After that, membrane was exposed to enhanced chemiluminescence (ECL) solution (Cytiva Lifescience™), and the signals were detected on ChemiDoc Imaging Systems (Bio-Rad). Primary antibodies were purchased from Cell Signaling Technology for EI24 (#42328), ASS1 (#70720), ARG2 (#55003), eNOS (#32027), pAKT (Ser473) (#9271), AKT (#9272), MDM2 (#86934), pP70S6K (#9204), P70S6K (#9202), pS6 (#2211), LC3 (#4108), and p21 (#2947); from Santa Cruz for ODC1 (sc-390366), ASL (sc-374353), and β-actin (sc-69879); from Novus for HIF1α (NB100-449); and from Sigma-Aldrich for puromycin (MABE343). Secondary antibodies horseradish peroxidase (HRP)-conjugated anti-mouse immunoglobulin G (IgG) (A16066) and anti-rabbit IgG (G212234) were from Invitrogen.

### Co-immunoprecipitation (co-IP)

Cells was lysed in Pierce™ IP lysis buffer (Thermo Scientific™) containing protease inhibitors (Sigma) and the phosSTOP (Roche) phosphatase inhibitor cocktail. Equal amounts of protein in each 500 μL of cell lysis were incubated overnight with 2 μL of puromycin or IgG antibody then 80 μL of Pierce™ protein A/G agarose (Thermo Scientific™) for 2 h at 4 °C. Beads were washed five times by rotation and centrifugation with lysis buffer. Then, 2× SDS loading buffer was used to elute the protein for western blot.

### Cell viability assay

Treated cells were stained with Crystal Violet solution containing Crystal Violet (C6158, Sigma) and paraformaldehyde in phosphate-buffered saline (PBS). Cell images were captured before dissolving Crystal Violet stains in methanol. The colorimetric absorbance was then measured at a wavelength of 570 nm.

### Scratch assay

MDA-MB-231 cells were seeded and grown to confluence in 12-well cell culture plates. Scratching of the monolayer of cells was done by using sterilized plastic tips before treatment. Cell images were captured immediately after the scratching and after 24 h and 48 h under a microscope (Evos). Invasive distance was measured by using ImageJ software [44].

### ASS1 activity assay

The ASS1 activity assay was carried out as previously described [28]. Cells were washed with PBS and then lysed in buffer A (50 mM Tris-HCl (pH 8), 10% glycerol, protease inhibitor cocktail). The reaction was started upon addition of reaction buffer (20 mM Tris-HCl (pH 7.8), 2 mM adenosine 5′-triphosphate, 2 mM citrulline, 2 mM aspartate, 6 mM MgCl_2_, 20 mM KCl, and 0.1 U pyrophosphatase) to the protein solution. The phosphate concentration in the product, indicative of ASS1 activity, was measured using molybdate buffer (10 mM ascorbic acid, 2.5 mM ammonium molybdate, and 2% sulfuric acid), which forms colorimetric signals read at 660 nm wavelength.

### Nitric oxide (NO) production assay

NO production represented by nitrite amount was measured using the nitric oxide synthase (NOS) activity assay kit (Colorimetric) (K205-100, Biovision) according to the manufacturer’s instructions. Briefly, cell lysate was incubated for reaction at 37 °C for 1 h. After incubation, an equal amount of assay buffer was added to each well to release nitrite, indicative of NO production, and then measured using Griess reagents, which produce a color detectable at a wavelength of 540 nm. Each experiment included a standard curve generated with nitrite standards.

### Polysome fractionation analysis

Polysome fractionation was performed by following the protocol outlined in a previous publication [45]. In brief, cells were lysed in polysome extraction buffer (10 mM Tris-HCl (pH 7.5), 100 mM KCl, 5 mM MgCl_2_, and 0.5% Nonidet P-40). Cell lysates with equal protein concentration were loaded onto columns of 10–50% sucrose gradient. Polysomes were separated by ultracentrifugation for 90 min at 39,000 RPM using a P40ST swinging bucket rotor. mRNA from the manually collected polysome fractions was extracted using TRIzol reagent and then transcripted into cDNA with ReverTra Ace™ qPCR RT Master Mix with gDNA Remover (Toyobo) for qPCR. The percentage of mRNA expression in each fraction was determined using the ΔCt method, with fraction 1 as the control. β-Actin (ACTB) percentage was included as a control.

### Surface sensing of translation (SUnSET) assay

The SUnSET assay is a widely established, nonradioactive method to assess protein synthesis rate [46]. Briefly, after treatment, cells were incubated in culture media containing 10 µM puromycin for 10 min then in fresh media for the next 50 min. Cells were then harvested in RIPA lysis buffer or Pierce™ IP lysis buffer (Thermo Scientific™) both containing protease and phosphatase inhibitors for either western blot or Co-IP experiments, respectively.

### In silico analysis

Gene set enrichment analysis (GSEA) was conducted to correlate the gene expression of EI24 and the enrichment of the arginine metabolism pathway in the breast cancer samples of datasets obtained from the Gene Expression Omnibus (https://www.ncbi.nlm.nih.gov/geo/, GEO accession nos. GSE50948 and GSE5364). Samples were stratified into three groups of low, medium, and high expression of EI24 on the basis of the first and third quartiles of expression before GSEA. Comparison of low and high phenotypes was performed by using GSEA software (http://www.broad.mit.edu/gsea/) [47, 48]. GSEA was also conducted on the WEB-based GEne SeT AnaLysis Toolkit (WebGestalt, https://www.webgestalt.org/) [49] to reveal the differential pathways in EI24 knockdown (GSE52508) or EI24 overexpression (GSE154422).

Gene expression of EI24 and ASS1 was obtained from OncoDB [50, 51] (http://oncodb.org/index.html) and graphed to compare between normal and breast cancer tissues.

The prognostic significance of EI24 and ASS1 was assessed by generating Kaplan-Meier plots using the Gene Expression database of Normal and Tumor tissues (GENT2) [52] (http://gent2.appex.kr/gent2/) or Gene Expression Profiling Interactive Analysis (GEPIA) [53] (http://gepia.cancer-pku.cn/) web servers. The median gene expression was used upon to classify patients into groups with high or low expression of that gene.

Levels of metabolites including arginine in breast cancer samples of the Tang et al. dataset were downloaded from the Cancer Atlas of Metabolic Profiles (CAMP, https://doi.org/10.5281/zenodo.7150252) [54] and matched with two groups of patients with low or high expression of EI24 as classified by the first and third quartiles of expression [55]. Outliers of arginine level were determined by using the interquartile range method.

### Statistical analysis

Student’s *t*-test was used to statistically compare means of two groups, while one-way analysis of variance (ANOVA) was used to test the difference among three or more groups. All graphs and statistical analysis were carried out by using GraphPad Prism version 10.0. Data are presented as mean ± SD (*n* ≥ 3, except where indicated), with *p-*values less than 0.05 considered significant. Asterisks indicate *p-*values as follows: * *p* ≤ 0.05, ** *p* ≤ 0.01, *** *p* ≤ 0.001, and **** *p* ≤ 0.0001.

## Results

### EI24 increased ASS1 upon arginine deprivation in ASS1-deficient breast cancer cells

**Fig. 1:**
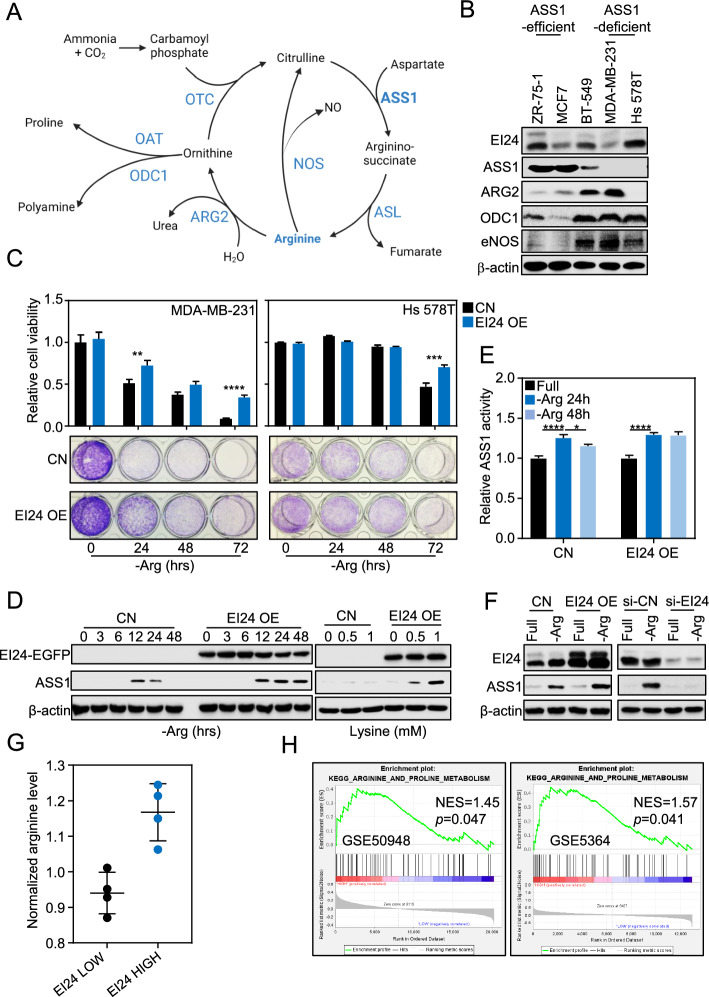
EI24 increased ASS1 upon arginine deprivation in ASS1-deficient breast cancer cells. **A** Urea cycle-related enzymes (CPS1, carbamoyl phosphate synthetase 1). **B** Expression of arginine metabolism enzymes in a subset of breast cancer cell lines. **C** Viability of breast cancer cells upon arginine deprivation with EI24 overexpression (OE) by doxycycline (MDA-MB-231) or adenovirus (Hs 578T). **D** Expression of ASS1 upon arginine deprivation (left) or lysine supplementation for 24 h (right) in MDA-MB-231 with EI24 OE by doxycycline. **E** Enzymatic activity of ASS1 upon time-dependent deprivation of arginine with EI24 OE by doxycycline in MDA-MB-231. **F** Expression of ASS1 upon arginine deprivation in 24 h in Hs 578T cells with EI24 OE by adenovirus (left) and knockdown by siRNA (right). **G** Arginine level in human breast cancer samples classified by EI24 gene expression. **H** Correlation of EI24 expression and Kegg_arginine_and_proline_metabolism pathway determined by GSEA in two different breast cancer datasets

To validate the importance of arginine in cancer growth, we exposed breast cancer cells of different subtypes including ZR-75-1 (luminal B), MCF7 (luminal A), and MDA-MB-231 (basal-like) [56] to culture media lacking one or several amino acid(s) and observed the diverse response to each condition. Notably, among these amino acids, arginine stood out as the most important for the survival of the most aggressive cells MDA-MB-231 [57] (Supplementary Fig. S1A). Consistently, blots of arginine metabolism proteins (Fig. 1A, B) showed that MDA-MB-231 and Hs 578T, were naturally deficient of arginine synthesis enzyme ASS1 yet increased arginine utilizing enzymes arginase 2 (ARG2), ornithine decarboxylase 1 (ODC1), and nitric oxide synthase (eNOS), indicating their higher demand while lacking de novo arginine synthesis capacity, hence being dependent on exogenous arginine (Fig. 1B). Next, to investigate whether EI24 is important for cancer cells during arginine deprivation, we overexpressed EI24 using doxycycline in MDA-MB-231 and adenovirus in Hs 578T. The results showed that overexpression (OE) of EI24 increased cancer cell viability in such conditions (Fig. 1C). Mechanistically, transient resurgence of ASS1 in ASS1-deficient cells as a response to arginine deprivation was prolonged by EI24 (Fig. 1D, left). Meanwhile, other important enzymes of arginine metabolism such as argininosuccinate lyase (ASL) and ARG2 remained unaffected by the presence of EI24 (Supplementary Fig. S1B). Such response of ASS1 was not seen in ASS1-efficient cells (Supplementary Fig. S1C). EI24 also induced ASS1 when we inhibited arginine transport by treating the cells with supraconcentration of arginine-competing amino acid lysine [58] (Fig. 1D, right). In parallel with protein expression, ASS1 enzymatic activity measured by phosphate released was also stabilized by EI24 (Fig. 1E). Harboring higher EI24 expression compared with MDA-MB-231, Hs 578T cells (Fig. 1B) survived longer after arginine deprivation (Fig. 1C) and similarly had ASS1 increased by EI24 overexpression and suppressed by silencing of EI24 using siRNA (Fig. 1F). Objectively, analysis of public breast cancer datasets revealed a considerably higher arginine level in cancer tissues harboring high expression of *EI24* (Fig. 1G), and EI24 linked to the enrichment of arginine_and_proline_metabolism (Fig. 1H), while gene expression data from OncoDB suggested an overall reduction of both ASS1 and EI24 in tumor compared with normal (Supplementary Fig. S1D). Taken together, in ASS1-deficient cancer, EI24 elevated ASS1 expression and activity thus sustained cell viability upon arginine depletion condition.

### EI24 regulates protein synthesis of ASS1

**Fig. 2:**
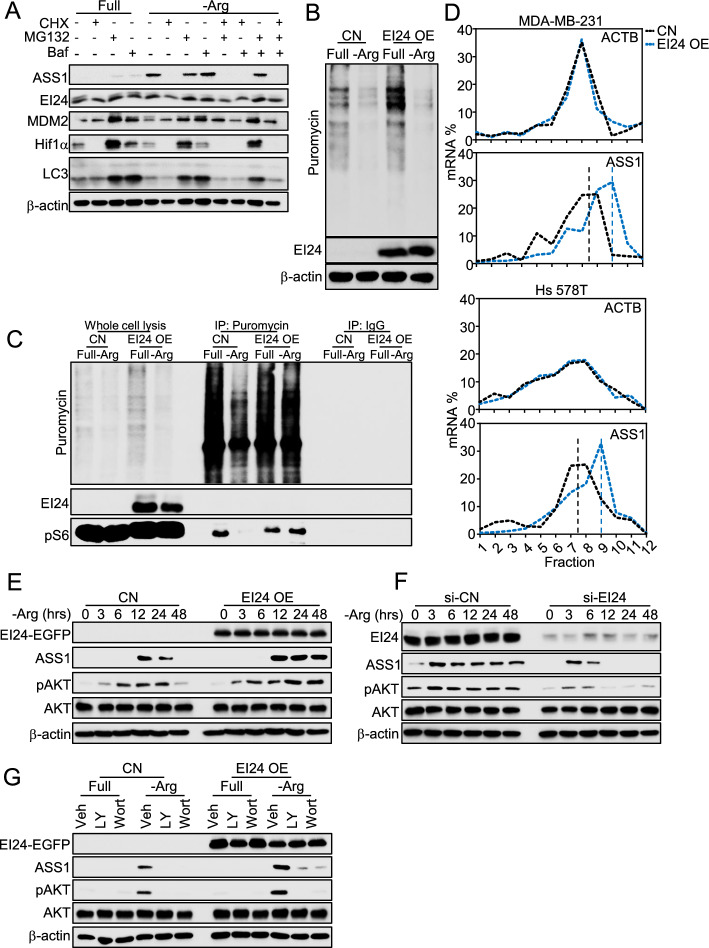
EI24 regulates protein synthesis of ASS1. **A** Expression of ASS1 upon arginine deprivation and/or cycloheximide (CHX, 10 µg/ml, 24 h), MG132 (10 µM, 6 h), and bafilomycin A1 (Baf, 10 nM, 2 h) treatment in MDA-MB-231 cells; murine double minute 2 (MDM2), hypoxia-inducible factor 1 subunit alpha (Hif1α), and microtubule-associated protein 1 A/1B-light chain 3 (LC3) were included as markers for treatment effects. **B** Global protein synthesis level with EI24 OE by adenovirus in MDA-MB-231 cells. **C** Integration of pS6 to ribosome upon EI24 OE by adenovirus in MDA-MB-231 cells examined by Co-IP. **D** ASS1 mRNA distribution analyzed by qPCR following polysome fractionation in MDA-MB-231 with EI24 OE by doxycycline (upper) and Hs 578T with EI24 OE by adenovirus (lower). **E** Phosphorylation of AKT upon time-dependent deprivation of arginine in MDA-MB-231 with EI24 OE by doxycycline. **F** Phosphorylation of AKT upon time-dependent deprivation of arginine in Hs 578T with knockdown of EI24 by siRNA. **G** ASS1 expression and phosphorylation of AKT upon arginine deprivation and/or PI3K inhibitors LY294002 (LY, 10 µM) and wortmannin (Wort, 50 µM) in 24 h in MDA-MB-231 with EI24 OE by doxycycline

We next sought to determine the mechanism by which EI24 modulates ASS1 expression. First, by qPCR analysis, we found that the mRNA level of ASS1 was elevated upon arginine deprivation, in both ASS1-deficient (Supplementary Fig. S2A), and ASS1-efficient (Supplementary Fig. S2B) cells, regardless of EI24 (Supplementary Fig. S2A). These data imply that EI24 enhanced ASS1 expression through post-transcriptional processes such as protein translation or degradation. Next, ASS1 emerged upon arginine deprivation was attenuated solely by protein translation inhibitor cycloheximide (CHX), but not proteasome inhibitor MG- 132, or autophagy inhibitor bafilomycin A (Baf) [27] (Fig. 2A), suggesting that ASS1 protein was mainly regulated by protein translation rather than degradation via proteosome or autophagy. To investigate direct involvement of EI24 in ASS1 translation, we performed SUnSET assay followed by western blot [46], which revealed a substantial gain of global protein synthesis upon EI24 overexpression (Fig. 2B). Note that arginine deletion quickly caused impairment of global protein synthesis (Supplementary Fig. S2C), which was not visually rescued by EI24 (Fig. 2B). However, co-IP analysis following SUnSET assay showed that EI24 maintained the incorporation of phosphorylation of ribosomal S6 protein to ribosome (Fig. 2C), which supposedly facilitates synthesis of critical proteins in such condition. Consistently, ribosome was among the most significantly downregulated or enriched pathways in EI24 silenced or overexpressed models, respectively (Supplementary Fig. S2D, E). Next, polysome fractionation analysis showed that EI24 specifically increased translation of ASS1 (Fig. 2D) as indicated by the distribution of ASS1 mRNA to larger polysomes, but not of other arginine metabolism enzymes (Supplementary Fig. S2F). The phosphoinositide 3-kinase (PI3K)/protein kinase B (AKT)/mammalian target of rapamycin (MTOR) axis is the mediator of protein translation [59], which was known to be transiently reactivated upon prolonged arginine deprivation [60]. Inhibition of MTOR indeed abolished arginine starvation-induced ASS1 (Supplementary Fig. S2G). Interestingly, that transient activation of AKT after depletion of arginine was stabilized by overexpression (Fig. 2E, Supplementary Fig. S2H) and suppressed by knockdown of EI24 (Fig. 2F). Inhibition of PI3K/AKT by wortmanin and LY29004 repressed ASS1 (Fig. 2G), further depicting the dependence of ASS1 on this axis. Taken together, when arginine was not available, EI24 elevated ASS1 mRNA translation in a PI3K/AKT-dependent fashion.

### Activation of PI3K/AKT-regulating ASS1 translation is through NO-regulated nitrosylation of PTEN

**Fig. 3:**
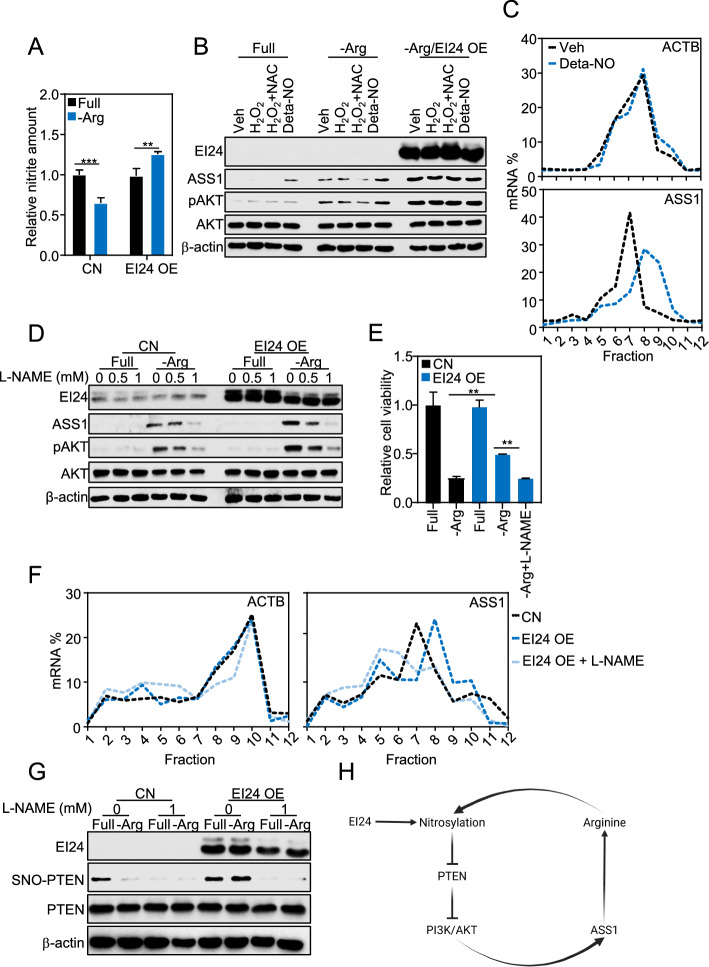
Activation of PI3K/AKT-regulating ASS1 translation is through nitric oxide (NO)-regulated nitrosylation of PTEN. **A** Relative nitrite amount in MDA-MB-231 cell lysis upon arginine deprivation with EI24 OE by adenovirus.** B** Expression of ASS1 and pAKT upon arginine deprivation and/or H_2_O_2_ and NO donor (deta-NO) treatment in MDA-MB-231 with EI24 OE by adenovirus (NAC, *N*-acetylcysteine). **C** ASS1 mRNA distribution in polysome fractions upon NO donor (deta-NO) treatment in MDA-MB-231. **D.** Effect of L-NAME on ASS1 and pAKT in MDA-MB-231 with EI24 OE by adenovirus. **E** Cell viability upon arginine deprivation and L-NAME treatment in MDA-MB-231 with EI24 OE by adenovirus. **F** ASS1 mRNA distribution in polysome fractions upon L-NAME treatment in MDA-MB-231 with EI24 OE by adenovirus. **G** *S*-nitrosylation of PTEN upon arginine deprivation and L-NAME treatment in MDA-MB-231 with EI24 OE by adenovirus. **H** Model of EI24-regulated translation of ASS1 via nitrosylation of PTEN

PI3K recruitment of AKT to the cell membrane for its activation can be blunted by phosphatase sequence homology to tensin (PTEN) [59], which in turn was inhibited selectively by nitric oxide (NO) through *S*-nitrosylation [61, 62]. Since arginine is the source of NO production (Fig. 1A), its depletion assumedly releases PTEN, leading to consecutive activation of AKT. Indeed, we found that exposure to arginine deprivation reduced the nitrite amount representing NO production in cancer cells, which was notably restored by EI24 (Fig. 3A). Accordingly, we hypothesized that EI24 persisted pAKT through inhibition of PTEN by NO. NO supplement DETA NONOate (deta-NO), but not reactive oxygen species H_2_O_2_, increased pAKT and ASS1 protein expression and translation (Fig. 3B, C). In contrast, inhibition of NO production by L-N^G^-nitro arginine methylester (L-NAME) blunted pAKT and ASS1 (Fig. 3D), leading to cell death (Fig. 3E) under arginine depletion. EI24-induced translation of ASS1 was also suppressed by L-NAME treatment (Fig. 3F). Meanwhile, biotin switch assay to detect nitrosylated proteins revealed that *S*-nitrosylated PTEN, which was reduced upon arginine deprivation, was sufficiently rescued by EI24 and dependent on NO production (Fig. 3G). Taken together, the regulation of EI24 on ASS1 translation likely occurs through releasing PI3K/AKT by nitrosylation of PTEN. This results in increased ASS1 expression, which helps maintain arginine levels essential for NO production, thereby establishing a vicious cycle of cancer response under arginine deprivation (Fig. 3H).

### Arginine is pivotal for polyamine synthesis in breast cancer

We next questioned the critical role of arginine that drives the rapid response of cancer cells to its depletion. Arginine can undergo conversion into urea, polyamine, or proline via the essential functions of ARG2, ODC1, or ornithine aminotransferase (OAT), respectively. qPCR data revealed that the mRNA expression of polyamine synthesis enzyme ODC1 considerably increased when arginine was omitted, while the other enzymes did not show similar changes (Supplementary Fig. S2A, S2B). This specified polyamine, a well-known factor of cancer aggression [15], as the major downstream and may substitute arginine for cancer demand. To elucidate this notion, spermidine, a polyamine, was supplemented to cells following arginine deprivation. As expected, treatment with spermidine rescued cell viability (Supplementary Fig. S3A, S3B) and cell invasion (Supplementary Fig. S3C, S3D) upon arginine depletion, similarly to overexpression of EI24, in cells lacking EI24. Polyamine treatment restored arginine deprivation-induced ASS1 expression to baseline (Supplementary Fig. S3E), while inhibition of polyamine synthesis enzyme ODC1 by difluoromethylornithine (DFMO) upregulated the ASS1 mRNA level (Supplementary Fig. S3F), together suggesting that cancer cells regulated arginine synthesis in accordance with the availability of polyamine.

### EI24 enhanced ASS1 expression in various ASS1-deficient cancers, independent of p53 as a transcription factor for ASS1

**Fig. 4:**
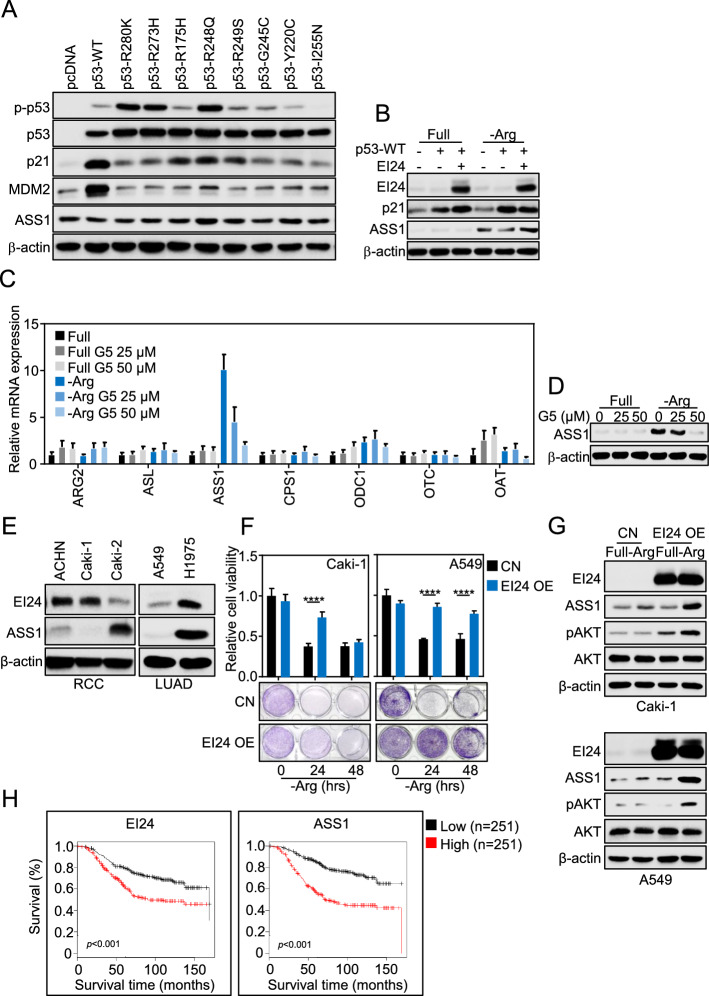
EI24 enhanced ASS1 expression in various ASS1-deficient cancers, independent of p53 as a transcription factor for ASS1. **A** Expression of ASS1 in p53-null H1299 upon transfection of wildtype or point-mutated p53 plasmids; p21 and MDM2 were included as positive control for p53 transfection. **B** Expression of ASS1 upon arginine deprivation in p53-mutated MDA-MB-231 with wildtype p53 plasmid transfection and EI24 OE by adenovirus. **C** mRNA expression of urea cycle-related enzymes upon arginine deprivation and/or 10074-G5 (G5) treatment in 24 h in MDA-MB-231. **D** Expression of ASS1 upon arginine deprivation and/or 10074-G5 (G5) treatment in 24 h in MDA-MB-231. **E** ASS1 expression in a subset of renal cell carcinoma (RCC) and lung adenocarcinoma (LUAD) cells. **F** Cell viability upon time-dependent arginine deprivation in Caki-1 and A549 with EI24 OE by adenovirus. **G** Expression of ASS1 and pAKT upon arginine deprivation for 24 h in Caki-1 and A549 with EI24 OE by adenovirus. **H** Prognostic value of EI24, and ASS1 in breast cancer determined by survival analysis on GENT2

Both EI24 and ASS1 were targets of wildtype p53 [28, 33]. Indeed, our breast cancer panels showed that p53-wildtype cells have efficient ASS1, as opposite to p53-mutated ones (Fig. 1B) [63], suggesting p53 as the transcription factor of ASS1. However, we transfected various p53 plasmids to p53-null H1299 lung cancer cells and found that both wildtype and mutated p53 that are impaired in canonical transcription activity [64] did not affect ASS1 expression (Fig. 4A, Supplementary Fig. S4A). Similarly, ASS1 expression remained resilient in p53-wildtype MCF7 regardless of arginine deprivation and/or mutated p53 presence (Supplementary Fig. S4B). In addition, induction of wildtype p53 to p53-mutated MDA-MB-231, though functionally promoted p21, did not increase ASS1 any further than EI24 did (Fig. 4B, Supplementary Fig. S4C). We thus proposed another potential transcription driver c-MYC [29–31], as it was stimulated upon arginine deprivation [65] (Supplementary Fig. S4C). Treatment with 10074-G5, a small-molecule inhibitor of c-MYC transcriptional activity, suppressed ASS1 resurgence transcriptionally in MDA-MB-231 (Fig. 4C, D). Meanwhile, induction of Hif1α by deferoxamine or CoCl_2_, known to repress ASS1 transcription elsewhere [32], did not affect ASS1 mRNA induced under arginine deprivation (Supplementary Fig. S4D). Lastly, to validate our finding in other types of cancer, ASS1 expression was assessed in subsets of renal cell carcinoma (RCC) and lung adenocarcinoma (LUAD), among which, Caki-1 and A549, even harbor wildtype p53 [66, 67], appeared to be ASS1-deficient (Fig. 4E). Nevertheless, EI24 substantially prolonged cancer cell survival upon arginine deprivation by enhancing ASS1 in these cell lines (Fig. 4F, G), but not ASS1-efficient cells (Supplementary Fig. S4E, F). Overall, EI24 seemed to sustain ASS1 translation regardless of the transcription factor driving ASS1 mRNA expression. With this tumor prosurvival role, EI24 and ASS1 both showed poor prognostic value in patients, not only in a same breast cancer dataset (Fig. 4H), but also across various cancer types (Supplementary Fig. S4G).

## Discussion

Alterations of the urea cycle toward maximizing the use of nitrogen for macromolecules rather than disposal lead to impairment of the arginine synthesis machinery in cancer [13], as illustrated by the downregulation of the rate-limiting enzyme ASS1 across multiple cancers [68, 69]. This ASS1 silencing conjointly benefits cancer cells in terms of DNA damage adaptation [70], releasing serine synthesis [71], and promoting chemotherapy resistance [72]. Nevertheless, it reveals an immense reliance of cancer cells on exogenous arginine, as the arginine level exclusively increases in patients’ plasma [73], creating a chance for treating cancer by starving it of arginine [20].

In agreement with other study showing reexpression of ASS1 in cancer tissues after treatment with arginine depletion enzyme ADI-PEG20 [74], on the basis of which cancer cells might acquire treatment resistance, we also observed the emergence of ASS1 in cancer cells lacking basal ASS1 upon being deprived of arginine in culture media. Notably, we pointed out that this appearance of ASS1 was transient, but could be prolonged by the presence of EI24, and parallel EI24 inhibition of PTEN via *S*-nitrosylation thus enhanced ASS1 translation regulated by PI3K/AKT. Regulation of the ASS1 protein level has been reported elsewhere, albeit rarely, as being inactivated through acetylation at K165 and K176 [75] or proteasome degradation [76]. The novel translational regulation of ASS1 provided in this study was dependent on NO production, which in turn requires availability of arginine [77], forming an interdependent NO-PI3K/AKT-ASS1-arginine-NO cycle. We demonstrated that EI24 facilitated this cycle through additionally sustaining the NO level, thereby preventing AKT compromise and facilitating ASS1 translation on the polysome. In a recent report, under oxidative stress condition, EI24 was also found to protect pancreatic beta cells by inhibiting translation of nicotinamide adenine dinucleotide phosphate (NADPH) oxidase 4 (NOX4) by attaching an RNA-binding protein to the 3′-UTR of NOX4. While the notion that EI24 promotes or inhibits protein translation seems to be context-dependent, both cases result in better cell adaptation to stresses. Similarly, while the functions of EI24 in cancer are still under debate [39–42], we suggest that EI24 helps cancer cells overcome exhausting conditions such as metabolic limitation, as in this study. Further studies should be conducted to see whether EI24 exerts this tumor-promoting role in other stress circumstances, such as hypoxia, genome instability, etc., which are well established as hallmarks of cancer. Furthermore, ASS1 could increase as a response not only to arginine deprivation but also to chemotherapy, independently of p53 [72]. In our study, EI24 regulated ASS1 translation in various ASS1-deficient cancer cells, regardless of p53’s function as an ASS1 transcription factor. It would be very interesting to obtain a comprehensive view of all ASS1-activating conditions and the corresponding role of EI24.

Arginine deprivation was widely depicted to affect cancer cells through inducing mitochondrial distress [78, 79], emphasizing function of arginine in energy production. In addition, here we aimed to explore the metabolic fate of arginine and found that arginine was likely utilized as a precursor for polyamine, which was crucial for cancer survival and invasion [80] in malignant breast cancer cells. When arginine was scarce, cancer seemed to activate polyamine synthesis to compensate. In accordance with our report, a study by Locke et al. proposed a combination of ASS1 silencing and polyamine synthesis inhibition for synthetic lethality in mesothelioma [81].

While the potential of arginine deprivation therapy has been overshadowed by acquired resistance in cancer, our study describes a novel resistance mechanism by which EI24 sustains the arginine synthesis enzyme ASS1 through translational regulation and sheds light on the tumor-promoting function of EI24 with regard to cancer metabolism.

## Supplementary Information

Additional file 1.

Additional file 2.

## Data Availability

Public datasets used in this manuscripts are indicated in the respective figure or the “Materials and methods” section. Raw data and materials will be provided upon request to the corresponding author.

## Abbreviations

ASS1: Argininosuccinate synthase 1
EI24: Etoposide-induced protein 2.4 homolog
Hif1α: Hypoxia-inducible factor 1 subunit alpha
ER: Endoplasmic reticulum
FBS: Fetal bovine serum
CHX: Cycloheximide
DFMO: Difluoromethylornithine
L-NAME: *N*-nitro-l-arginine methylester
qPCR: Quantitative polymerase chain reaction
Co-IP: Co-immunoprecipitation
NO: Nitric oxide
NOS: Nitric oxide synthase
SUnSET: Surface sensing of translation
GSEA: Gene set enrichment analysis
ARG2: Arginase 2
ODC1: Ornithine decarboxylase 1
eNOS: Epithelial nitric oxide synthase
OE: Overexpression
ASL: Argininosuccinate lyase
MDM2: Murine double minute 2
LC3: Microtubule-associated protein 1A/1B-light chain 3
PI3 K: Phosphoinositide 3-kinase
AKT: Protein kinase B
MTOR: Mammalian target of rapamycin
PTEN: Phosphatase sequence homology to tensin
NAC: *N*-acetylcysteine
OAT: Ornithine aminotransferase
VEGFA: Vascular endothelial growth factor A
RCC: Renal cell carcinoma
LUAD: Lung adenocarcinoma
NOX4: Nicotinamide adenine dinucleotide phosphate oxidase 4
