# Supplementary material for: Etoposide-induced protein 2.4 homolog promotes argininosuccinate synthase 1 and cancer cell survival upon arginine deprivation
Source: Cell Mol Biol Lett. 2025 Apr 19;30:52. doi: 10.1186/s11658-025-00726-6 (PMC12008907; doi:10.1186/s11658-025-00726-6)
Supplement: Supplementary file 1 — Additional file 1. [file 11658_2025_726_MOESM1_ESM.pdf]

Supplementary information

Etoposide-induced protein 2.4 homolog promotes argininosuccinate synthase 1 and cancer cell survival upon arginine deprivation

Vu T.A. Vo, Tran Nhat Le, Bui Thanh Thu, Han-Woong Lee, and Yangsik Jeong*

*Corresponding author. Email: yjeong@yonsei.ac.kr

The supplementary information contains 5 figures.

Figure S1. **A**. Cell viability of breast cancer cells upon amino acid deprivation. **B**. ASL and ARG2 expression upon time-dependent arginine deprivation in MDA-MB-231 with EI24 OE by doxycycline. **C**. ASS1 expression on MCF7 and BT-549 with EI24 OE by adenovirus. **D**. Expression of EI24 and ASS1 in human breast cancer samples downloaded from OncoDB.

Figure S2. **A**. mRNA expression of urea cycle-related enzymes upon arginine deprivation in MDA-MB-231 with EI24 OE by doxycycline and Hs 578T with EI24 OE by adenovirus. **B**. mRNA expression of urea cycle-related enzymes in ZR-75-1 and MCF7. **C**. Global protein synthesis level upon time-dependent arginine deprivation with EI24 OE by adenovirus in MDA-MB-231. **D, E**. Differentially regulated pathways in EI24 KD (knockdown, **D**) and EI24 OE (**E**) analyzed on WebGestalt. **F**. ARG2, ASL, and ODC1 mRNA distribution in polysome fractions of Hs 578T with EI24 OE by adenovirus. **G**. Expression of ASS1 and pP70S6K upon arginine deprivation and torin treatment in MDA-MB-231. **H**. Expression of pAKT upon time-dependent arginine deprivation in Hs 578T with EI24 OE by adenovirus.

Figure S3. **A**. Cell viability upon arginine deprivation and spermidine supplementation in MDA-MB-231 with EI24 OE by doxycycline. **B**. Quantification of cell viability in A. **C**. Scratch assay upon arginine deprivation and spermidine supplementation in MDA-MB-231 with EI24 OE by doxycycline. **D**. Quantification of invasive distance in C. **E**. ASS1 expression upon arginine deprivation and spermidine treatment in MDA-MB-231 with EI24 OE by doxycycline (upper) and Hs 578T with EI24 OE by adenovirus (lower). **F**. ASS1 mRNA expression upon DFMO treatment in MDA-MB-231.

Figure S4. **A**. ASS1 mRNA expression upon transfection of wildtype or point-mutated p53 plasmids in H1299. **B**. ASS1 expression upon arginine deprivation and transfection of wildtype or point-mutated p53 plasmids in p53-wildtype MCF7. **C**. c-MYC expression upon arginine deprivation in MDA-MB-231 with EI24 OE by adenovirus. **D**. mRNA expression of ASS1 in MDA-MB-231 after 24 hours of Hif1α by deferoxamine (250 μM) and $CoCl_2$ (1 μM). **E**. Cell viability upon time-dependent arginine deprivation in Caki-2 and H1975 with EI24 OE by adenovirus. **F**. Expression of ASS1 and pAKT upon arginine deprivation in 24 hours in Caki-2 and H1975 with EI24 OE by adenovirus. **G**. Prognostic value of EI24 and ASS1 in various cancer types analyzed on GEPIA.

Figure S5. Western blot raw data.

Figure S1

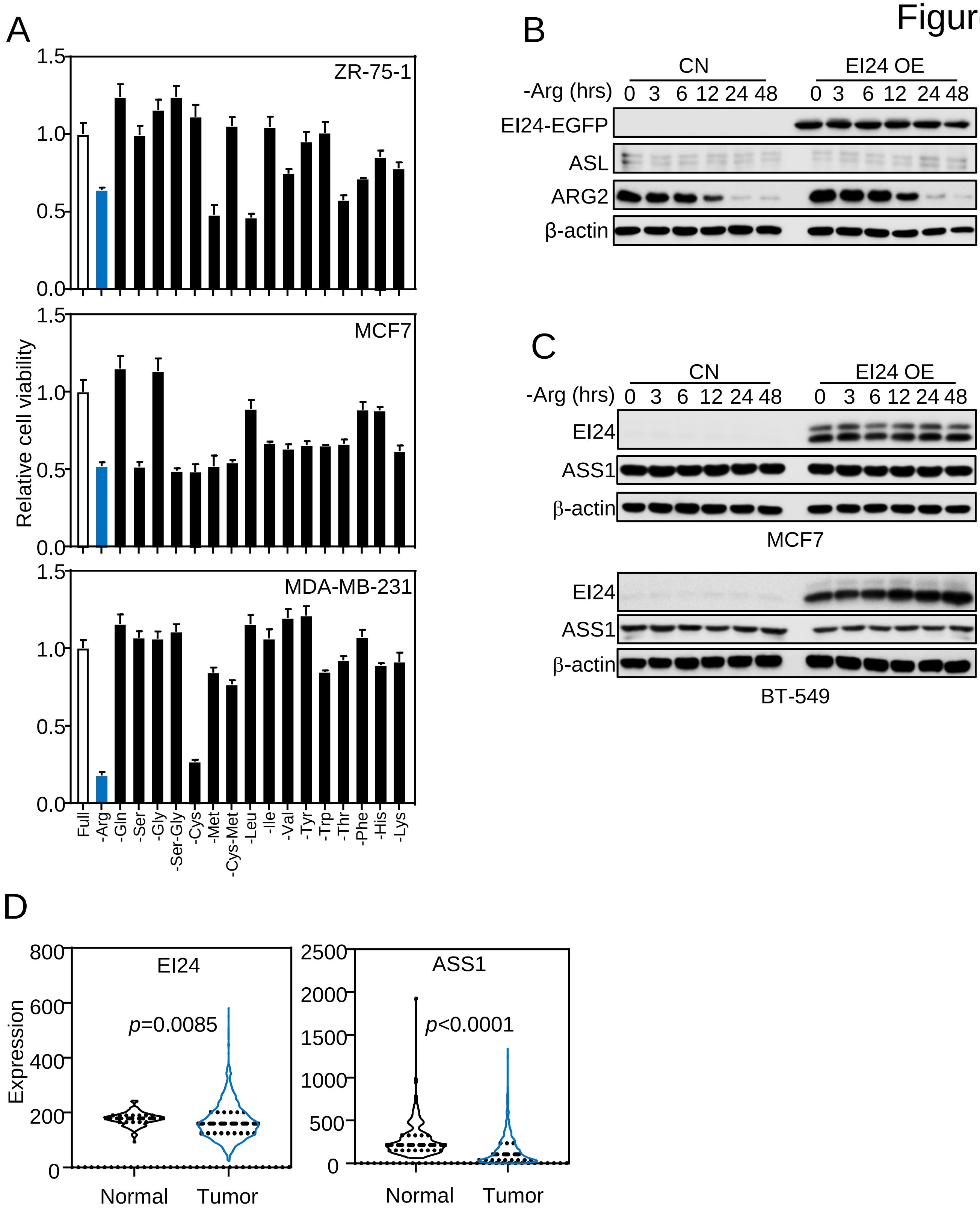
A
ZR-75-1
MCF7
MDA-MB-231
Relative cell viability
1.5
1.0
0.5
0.0
Full
-Arg
-Gln
-Ser
-Gly
-Ser-Gly
-Cys
-Met
-Cys-Met
-Leu
-Ile
-Val
-Tyr
-Trp
-Thr
-Phe
-His
-Lys
B
CN
EI24 OE
-Arg (hrs)
0 3 6 12 24 48
EI24-EGFP
ASL
ARG2
β-actin
C
EI24
ASS1
MCF7
BT-549
D
EI24
ASS1
Expression
p=0.0085
p<0.0001
Normal
Tumor
800
600
400
200
0
2500
2000
1500
1000
500

Figure S2

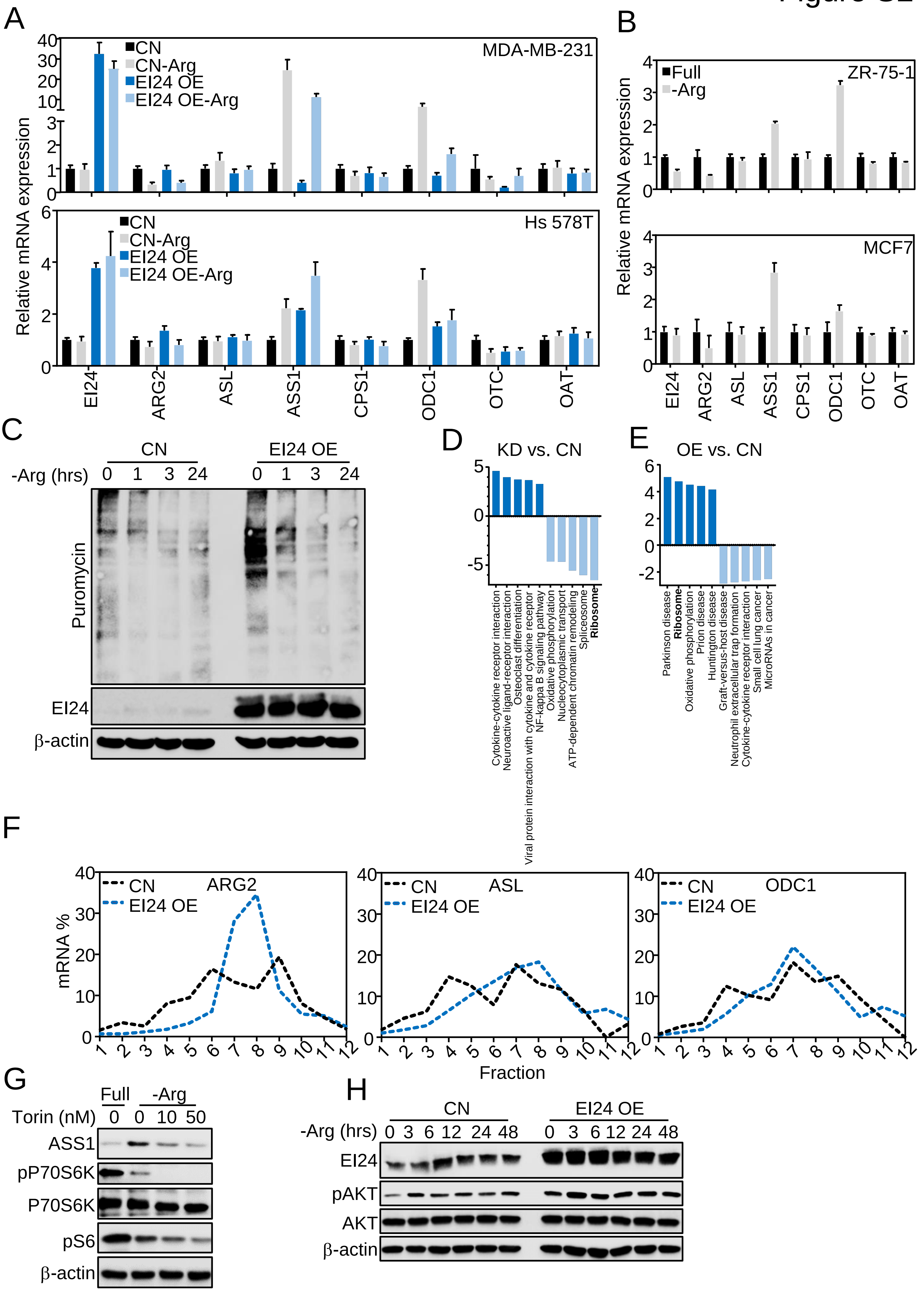
A
MDA-MB-231
CN
CN-Arg
EI24 OE
EI24 OE-Arg
Relative mRNA expression
Hs 578T
EI24
ARG2
ASL
ASS1
CPS1
ODC1
OTC
OAT
B
ZR-75-1
Full
-Arg
MCF7
C
CN
EI24 OE
-Arg (hrs)
0 1 3 24
Puromycin
EI24
β-actin
D
KD vs. CN
Cytokine-cytokine receptor interaction
Neuroactive ligand-receptor interaction
Osteoclast differentiation
Viral protein interaction with cytokine and cytokine receptor
NF-kappa B signaling pathway
Oxidative phosphorylation
Nucleocytoplasmic transport
ATP-dependent chromatin remodeling
Spliceosome
Ribosome
E
OE vs. CN
Parkinson disease
Ribosome
Oxidative phosphorylation
Prion disease
Huntington disease
Graft-versus-host disease
Neutrophil extracellular trap formation
Cytokine-cytokine receptor interaction
Small cell lung cancer
MicroRNAs in cancer
F
mRNA %
CN
EI24 OE
ARG2
ASL
ODC1
Fraction
G
Full
-Arg
Torin (nM)
0 0 10 50
ASS1
pP70S6K
P70S6K
pS6
β-actin
H
CN
EI24 OE
-Arg (hrs)
0 3 6 12 24 48
EI24
pAKT
AKT
β-actin

Figure S3

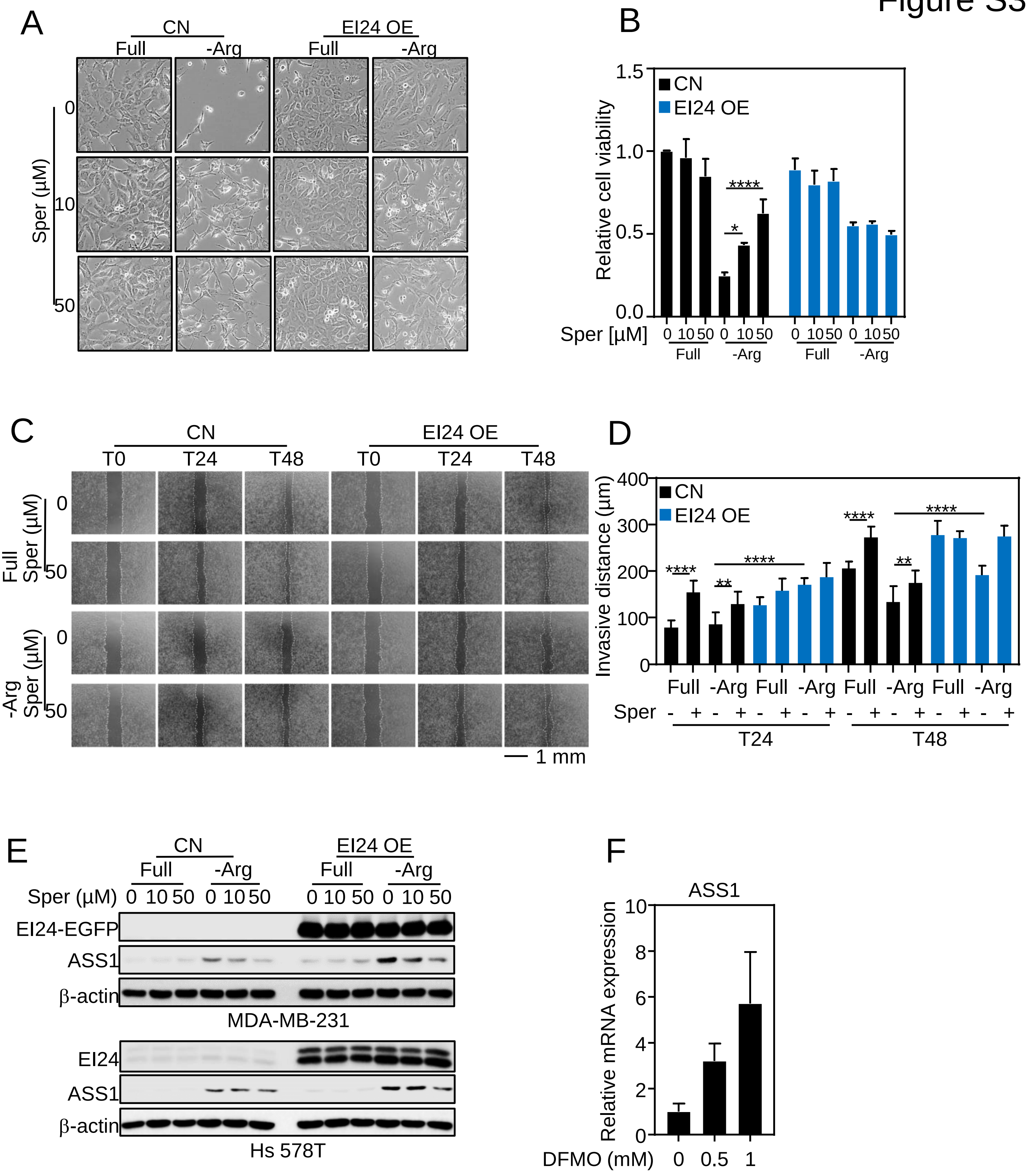
A
CN
EI24 OE
Full
-Arg
Sper (μM)
0
10
50
B
1.5
1.0
0.5
0.0
Relative cell viability
CN
EI24 OE
****
*
Sper [μM]
0 10 50 0 10 50 0 10 50 0 10 50
Full
-Arg
C
T0
T24
T48
Full
-Arg
1 mm
D
400
300
200
100
0
Invasive distance (μm)
**
Sper
T24
T48
E
EI24-EGFP
ASS1
β-actin
MDA-MB-231
EI24
Hs 578T
F
ASS1
10
8
6
4
2
Relative mRNA expression
DFMO (mM)
0.5
1

Figure S4

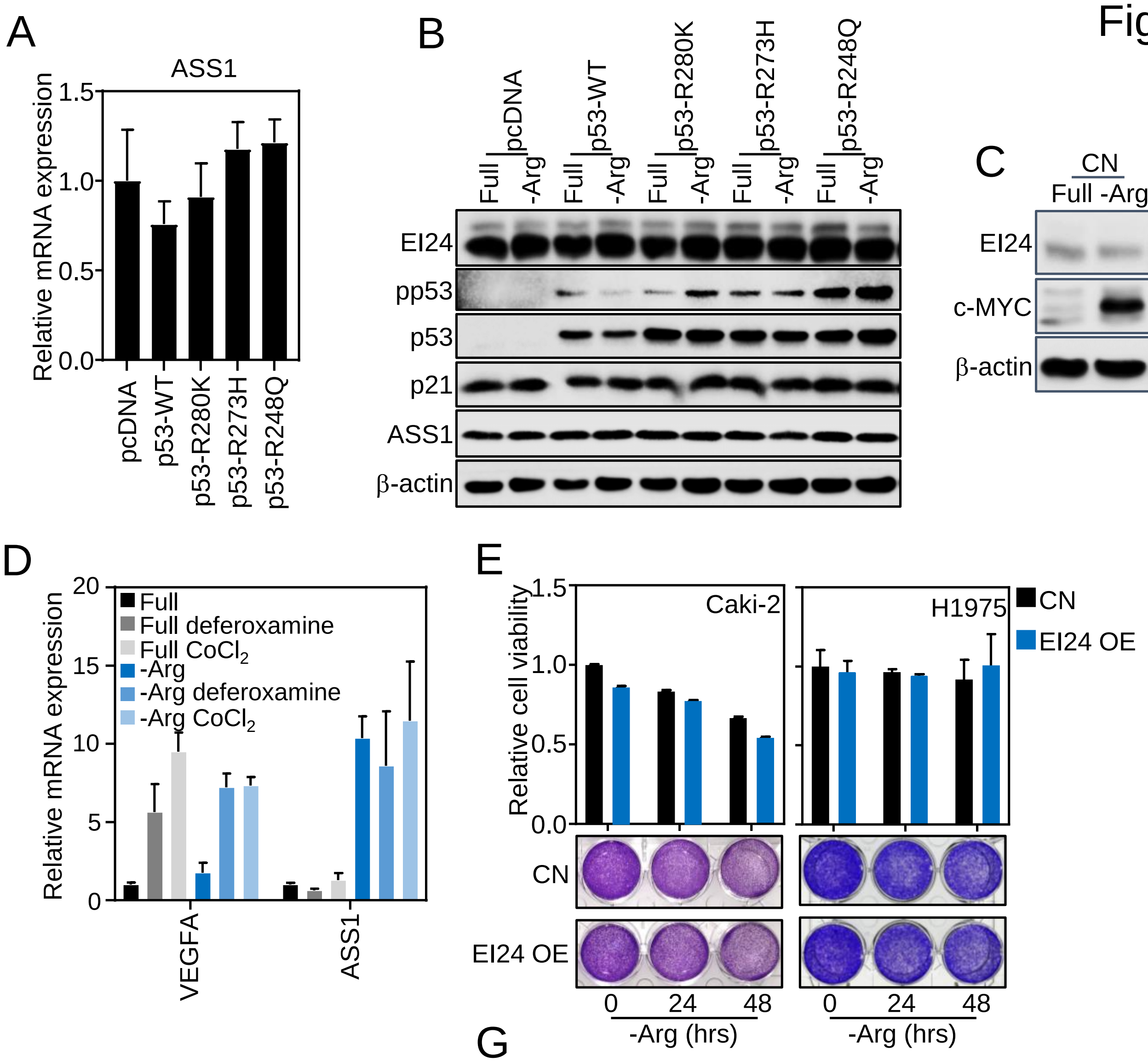

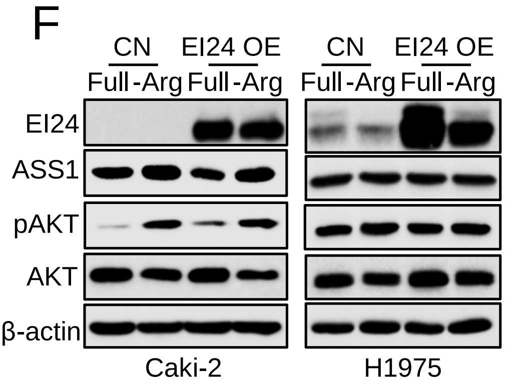

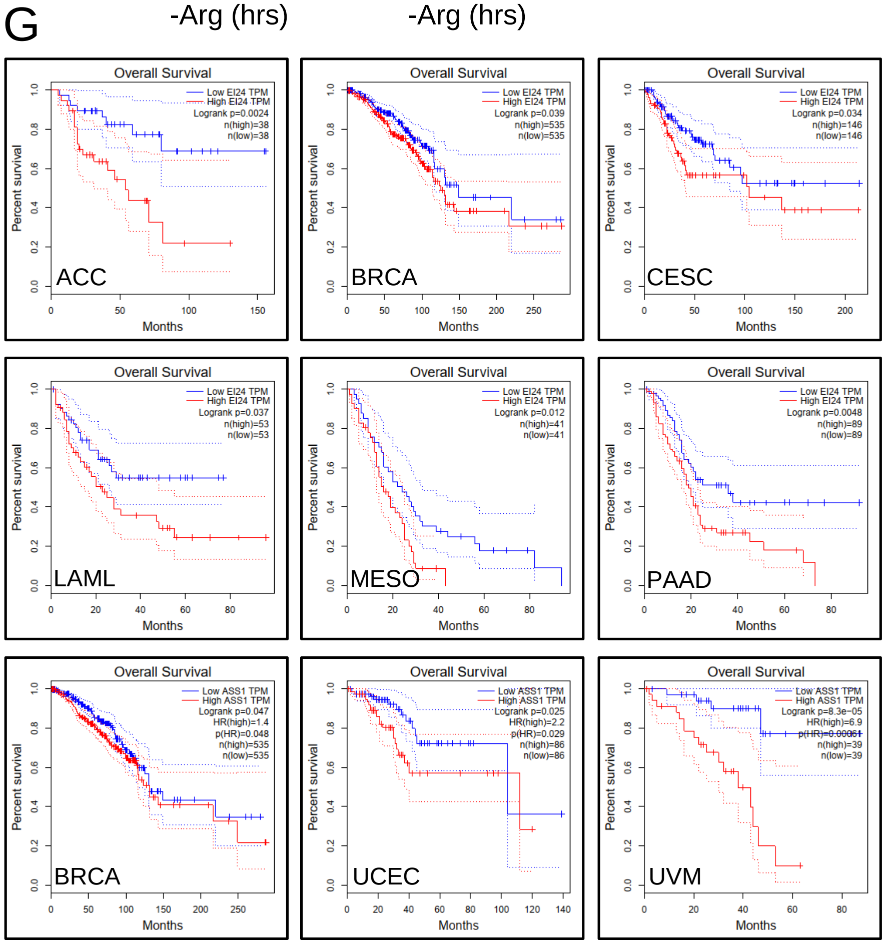

ACC, adrenocortical carcinoma
BRCA, breast invasive carcinoma
CESC, cervical squamous cell carcinoma and endocervical adenocarcinoma
LAML, acute myeloid leukemia
MESO, mesothelioma
PAAD, pancreatic adenocarcinoma
UCEC, uterine corpus endometrial carcinoma
UVM, uveal melanoma
