# Supplementary material for: Etoposide-induced protein 2.4 homolog promotes argininosuccinate synthase 1 and cancer cell survival upon arginine deprivation
Source: Cell Mol Biol Lett. 2025 Apr 19;30:52. doi: 10.1186/s11658-025-00726-6 (PMC12008907; doi:10.1186/s11658-025-00726-6)
Supplement: Supplementary file 2 — Additional file 2. [file 11658_2025_726_MOESM2_ESM.pdf]

Figure S5

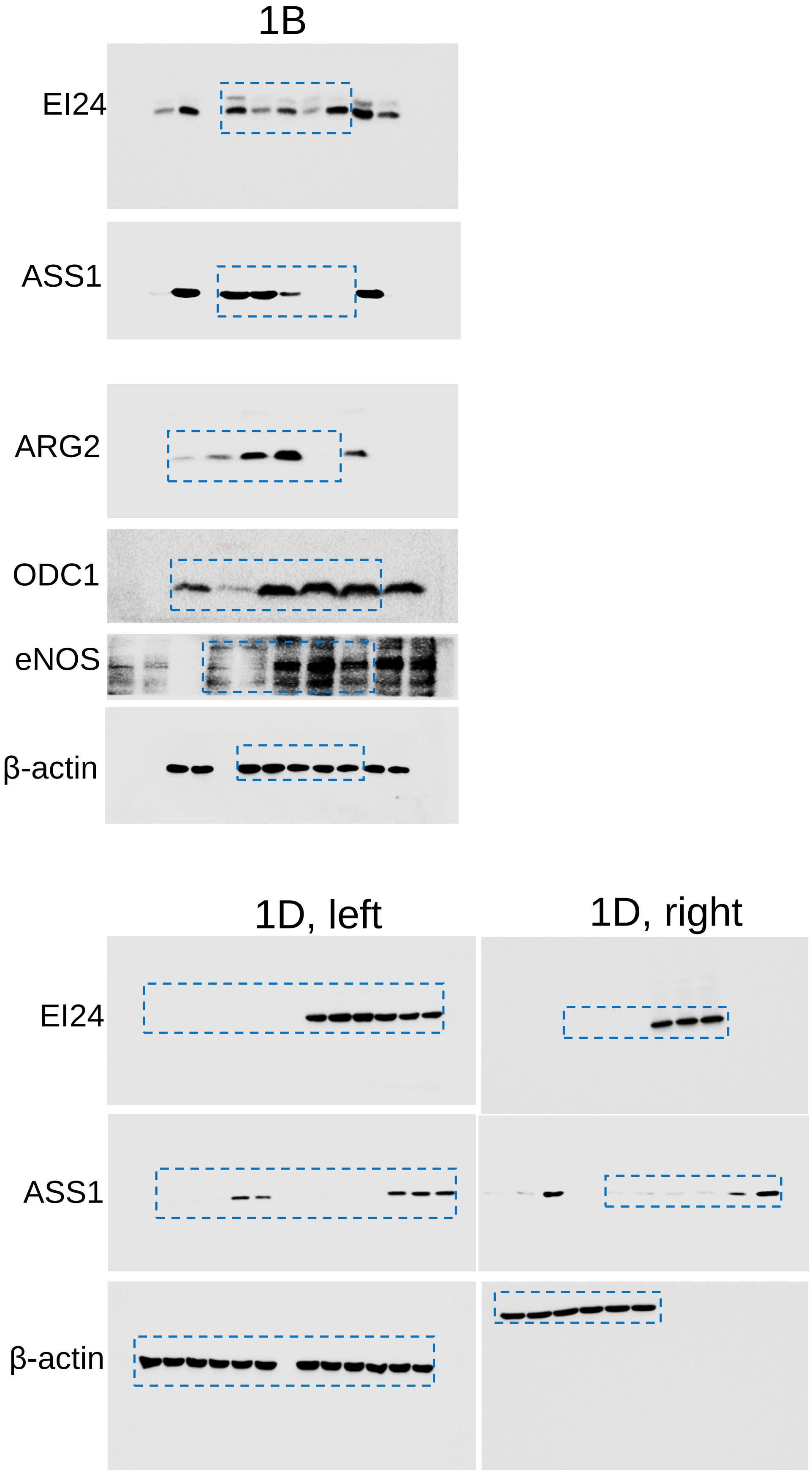
1B
EI24
ASS1
ARG2
ODC1
eNOS
β-actin
1D, left
1D, right
EI24
ASS1
β-actin

Figure S5

1F, left

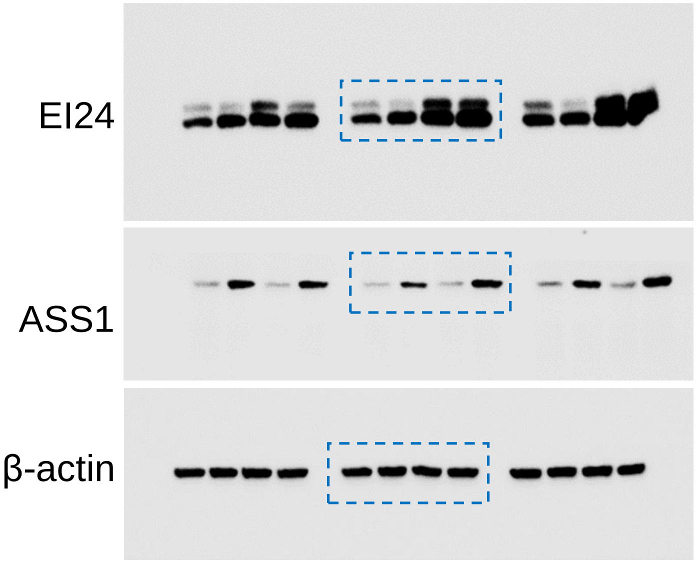

1F, right

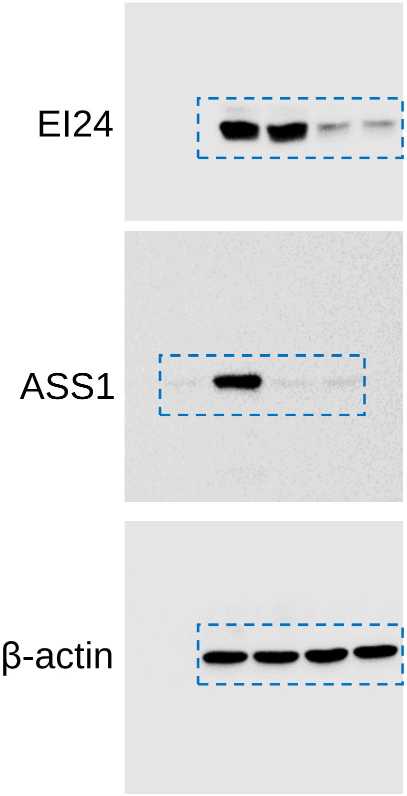

Figure S5

2A

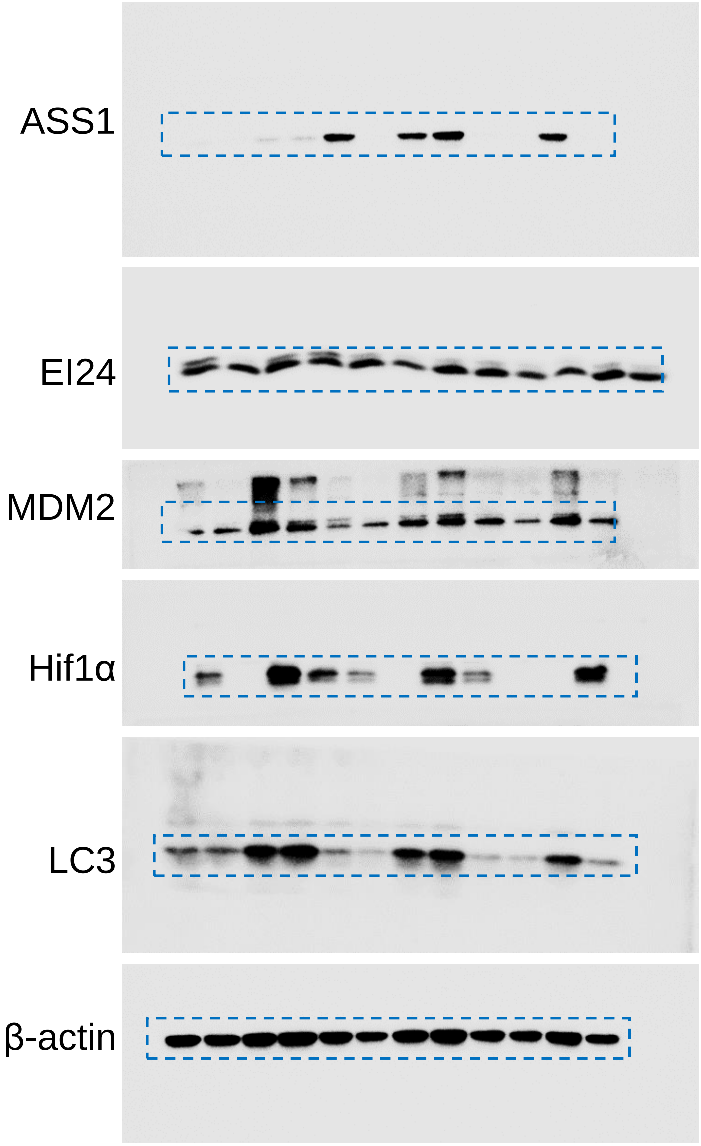

2B

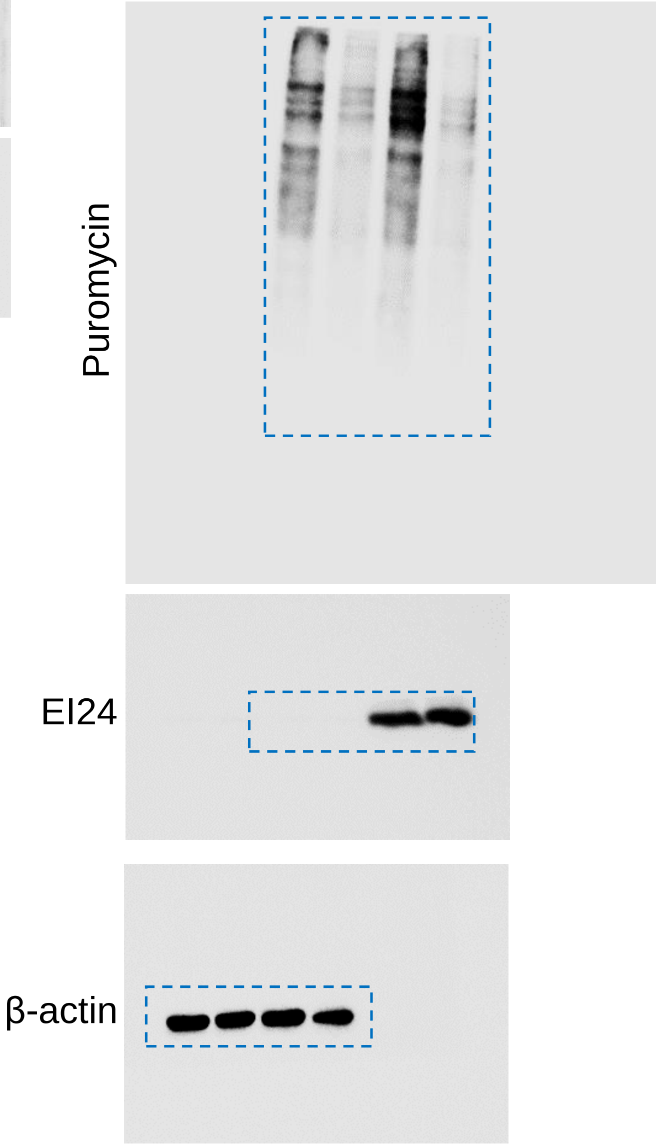

Figure S5

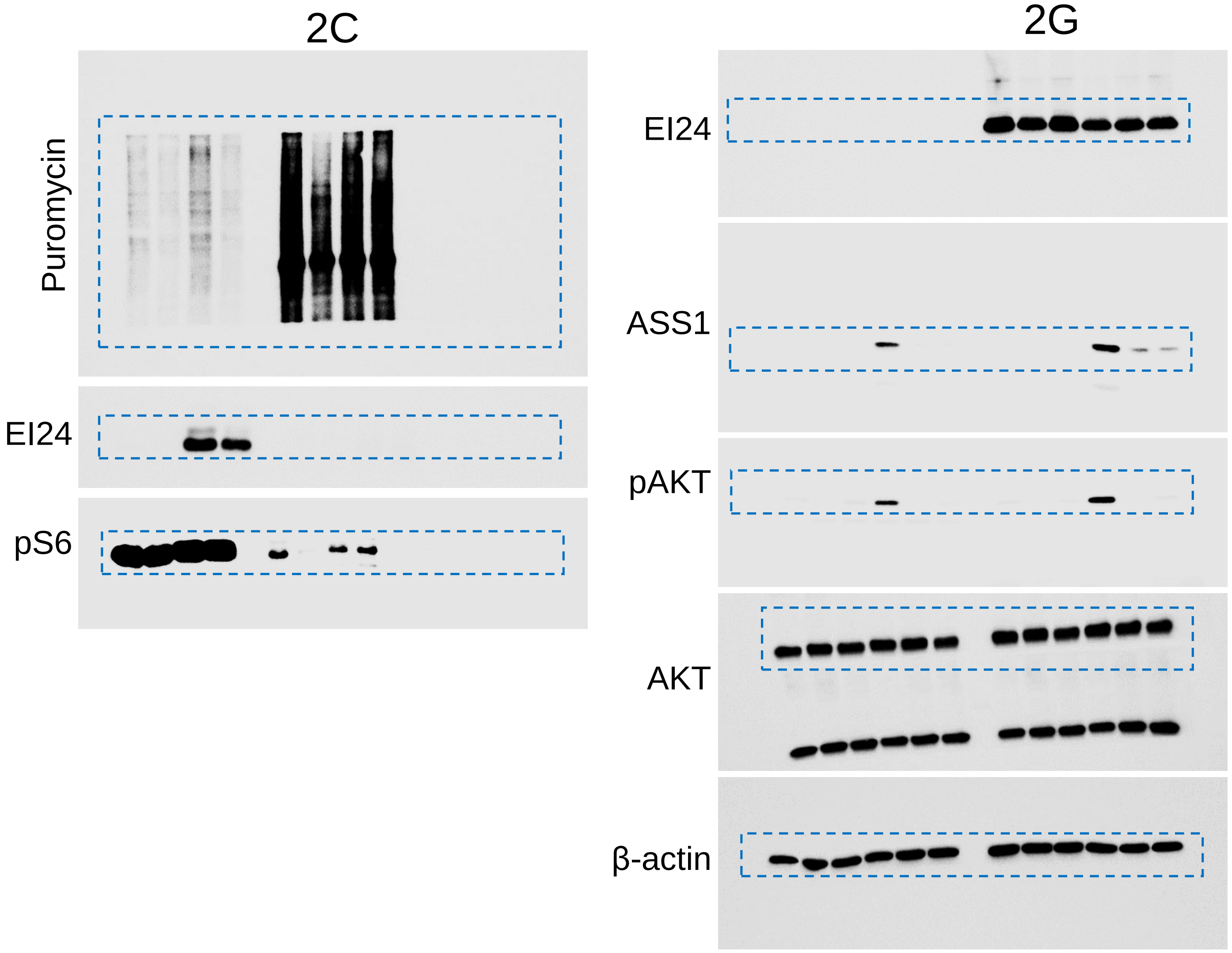

Figure S5

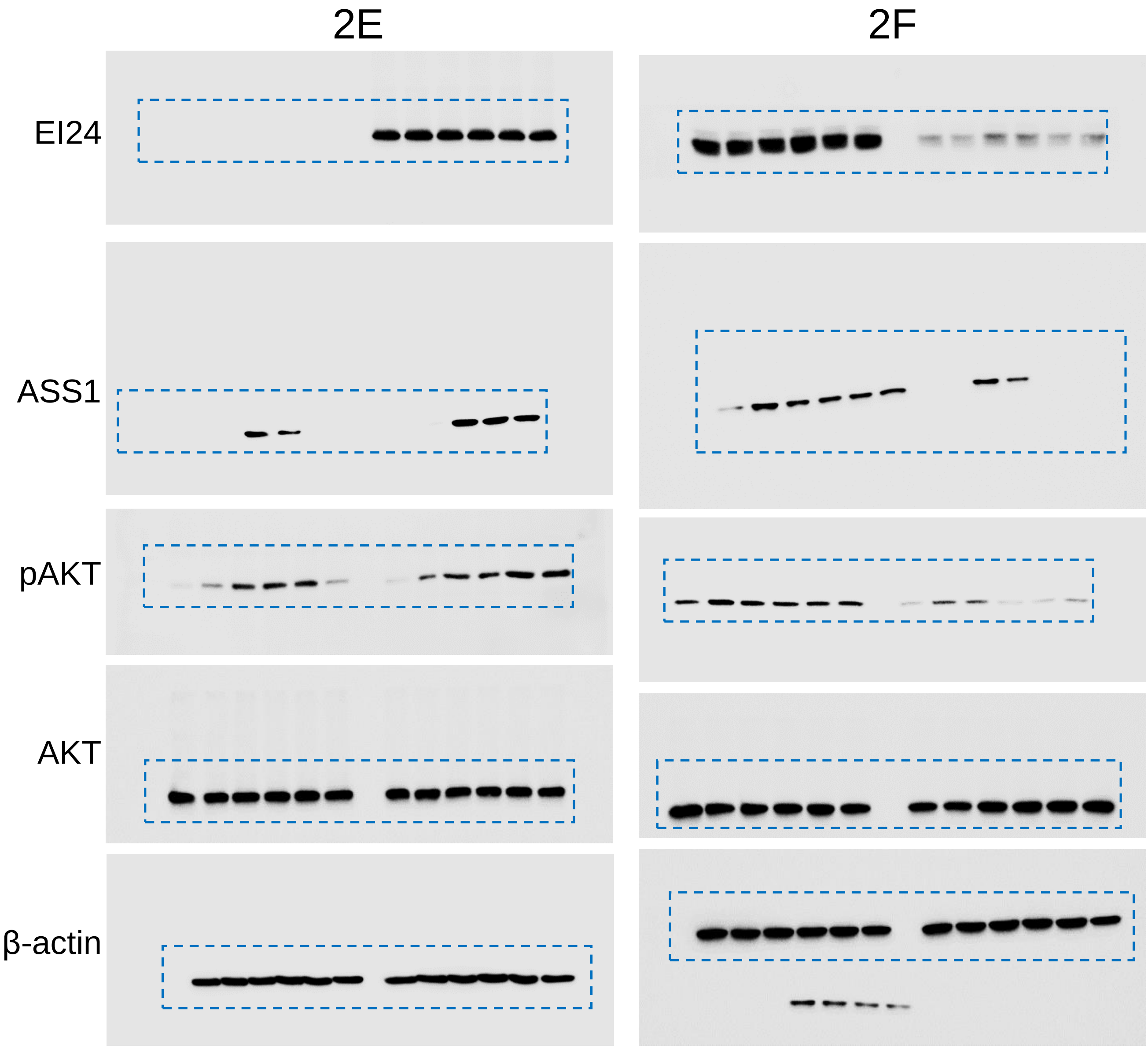

Figure S5

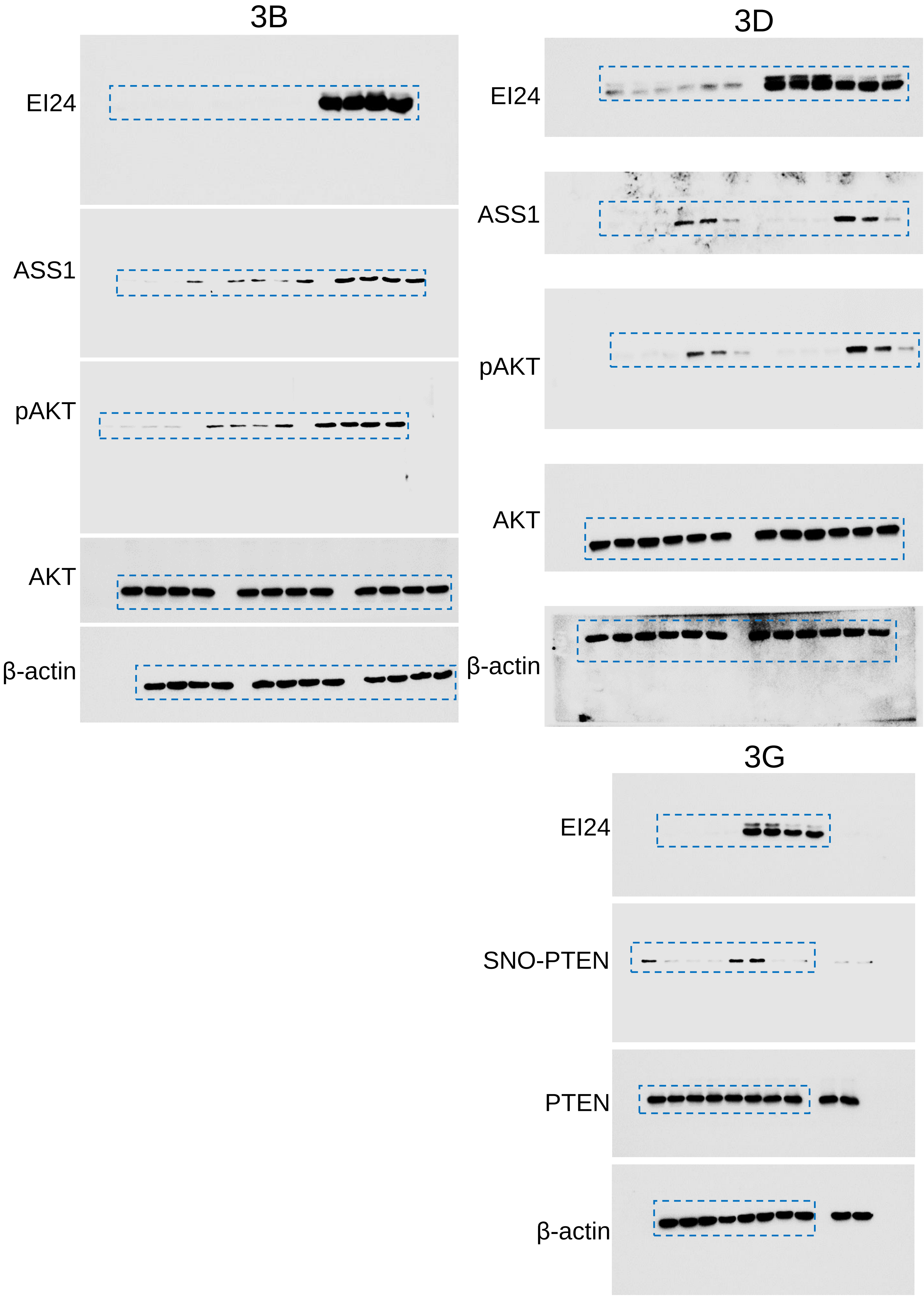
3B
EI24
ASS1
pAKT
AKT
β-actin
3D
EI24
ASS1
pAKT
AKT
β-actin
3G
EI24
SNO-PTEN
PTEN
β-actin

Figure S5

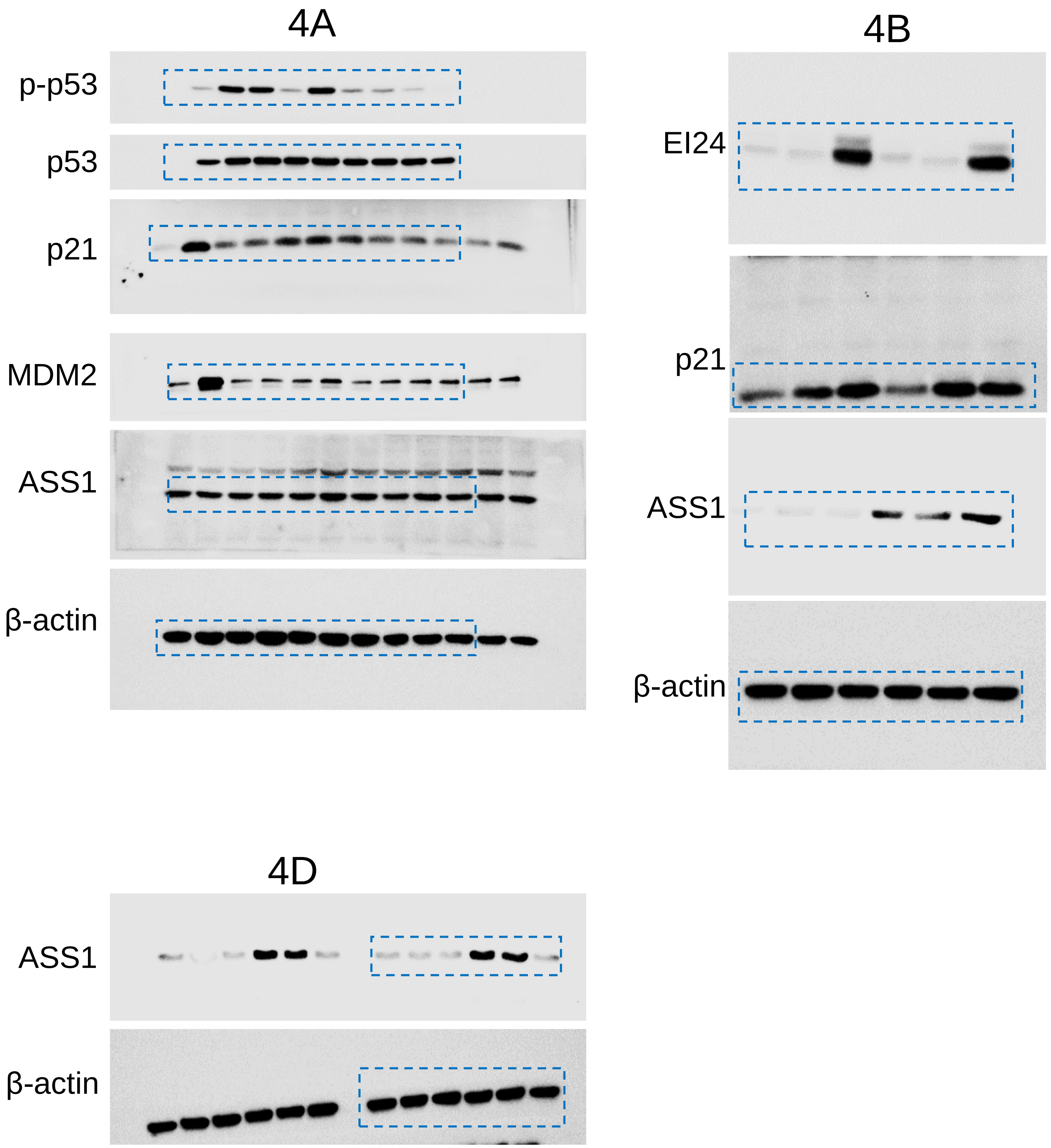
4A
p-p53
p53
p21
MDM2
ASS1
β-actin
4B
EI24
p21
ASS1
β-actin
4D
ASS1
β-actin

Figure S5

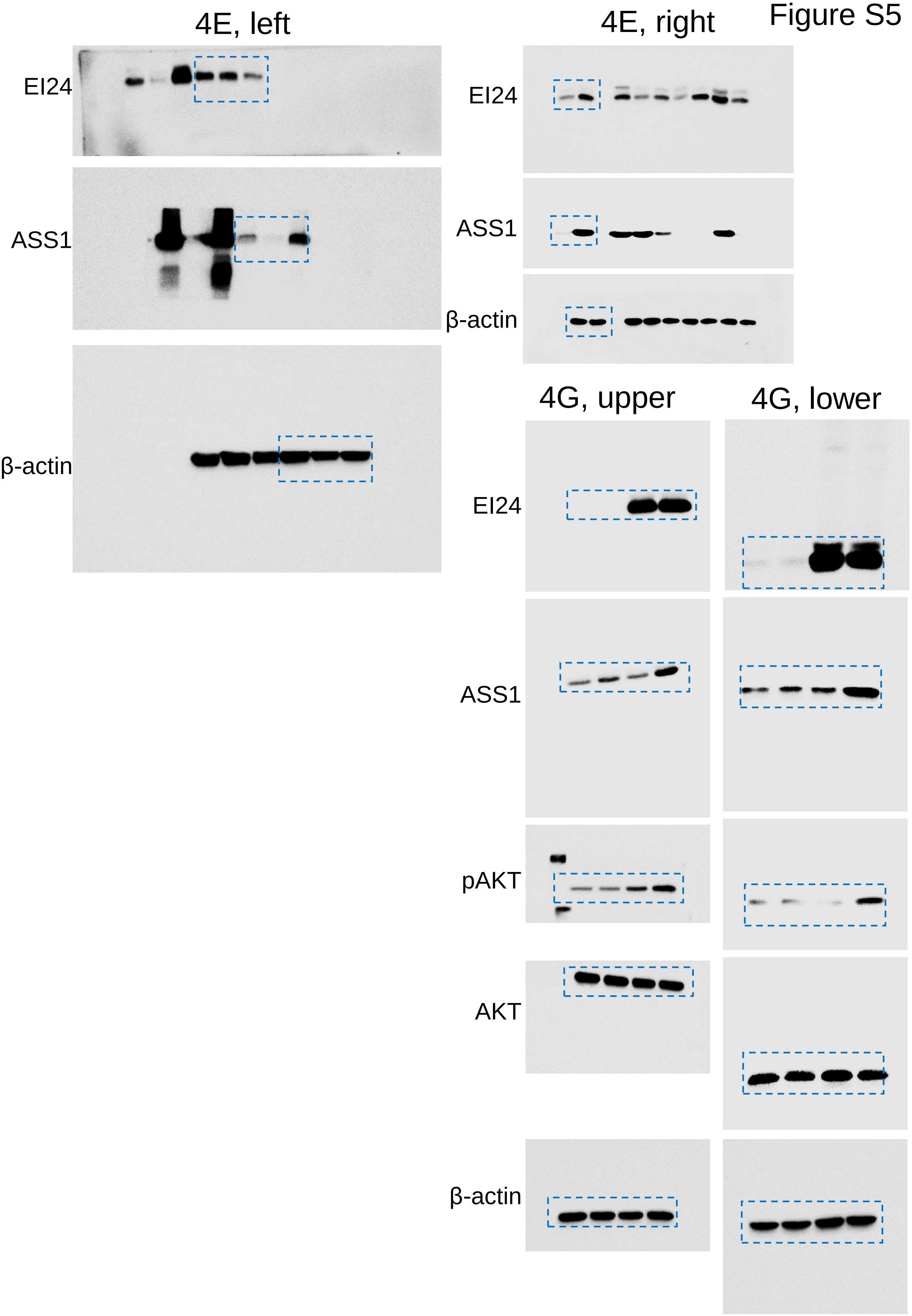
4E, left
EI24
ASS1
β-actin
4E, right
EI24
ASS1
β-actin
4G, upper
4G, lower
EI24
ASS1
pAKT
AKT
β-actin

Figure S5

S1B

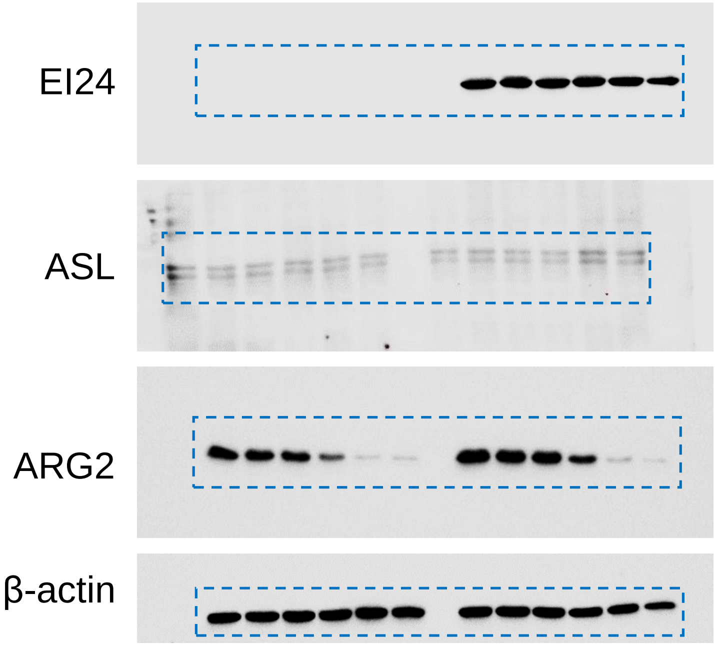

S1C, upper

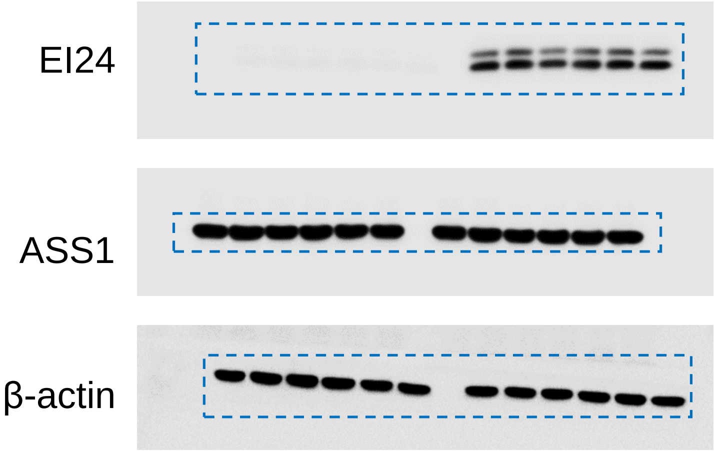

S1C, lower

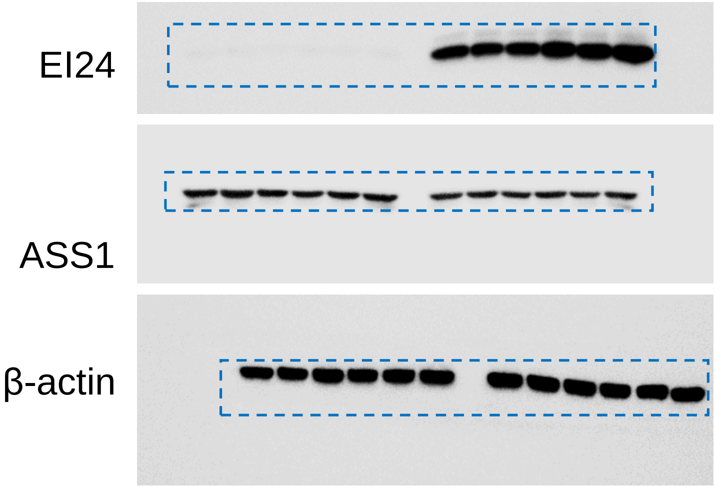

Figure S5

S2C

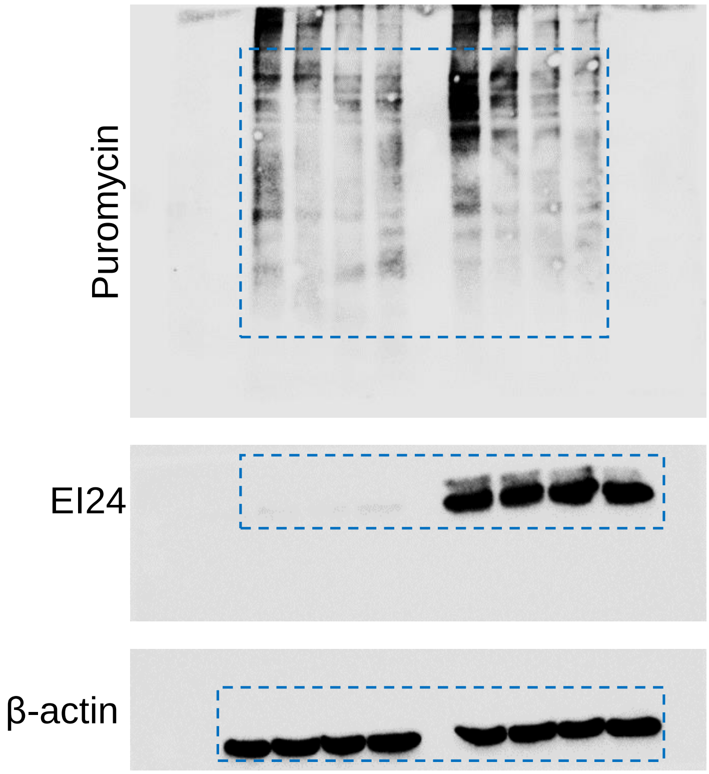

S2G

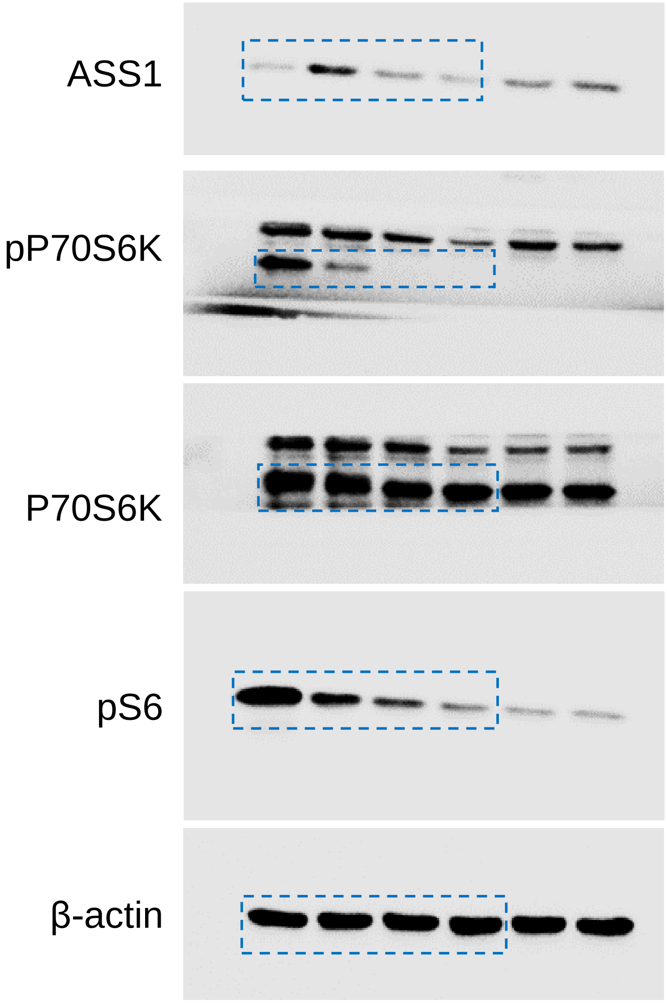

S2H

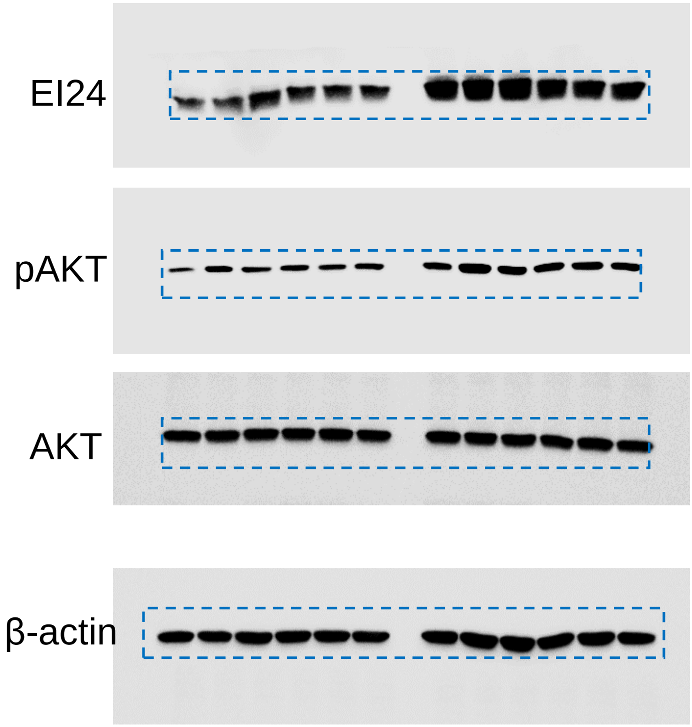

Figure S5

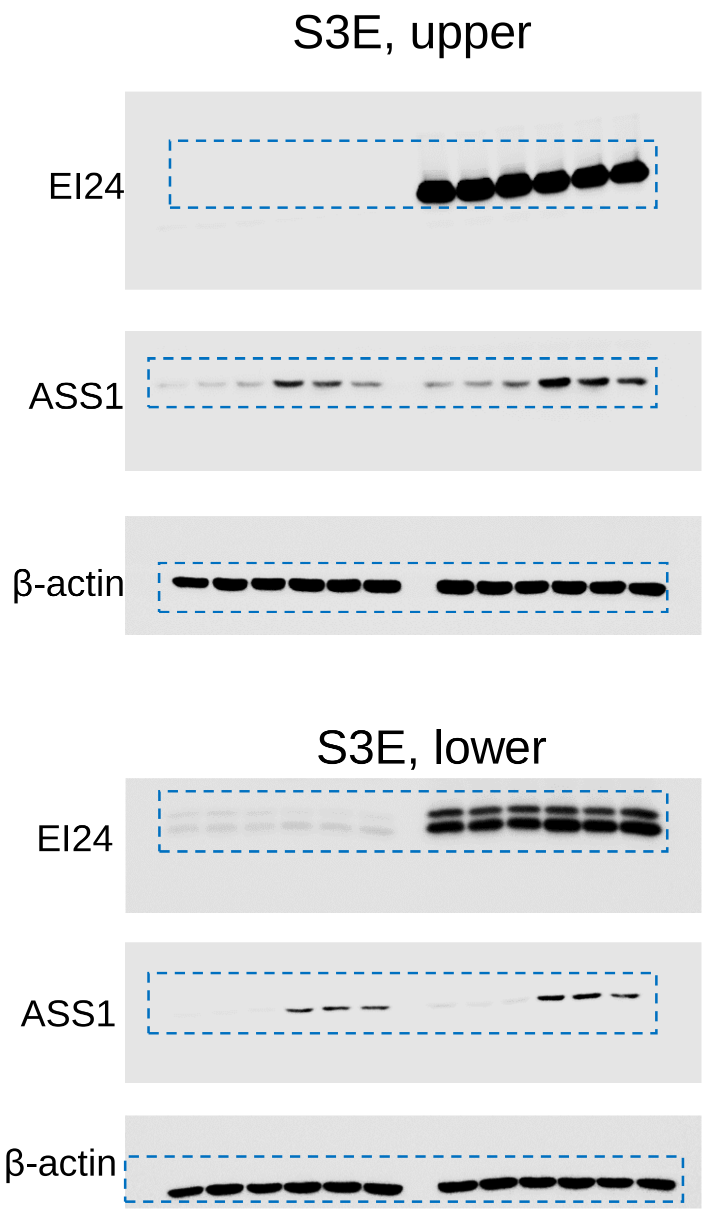

Figure S5

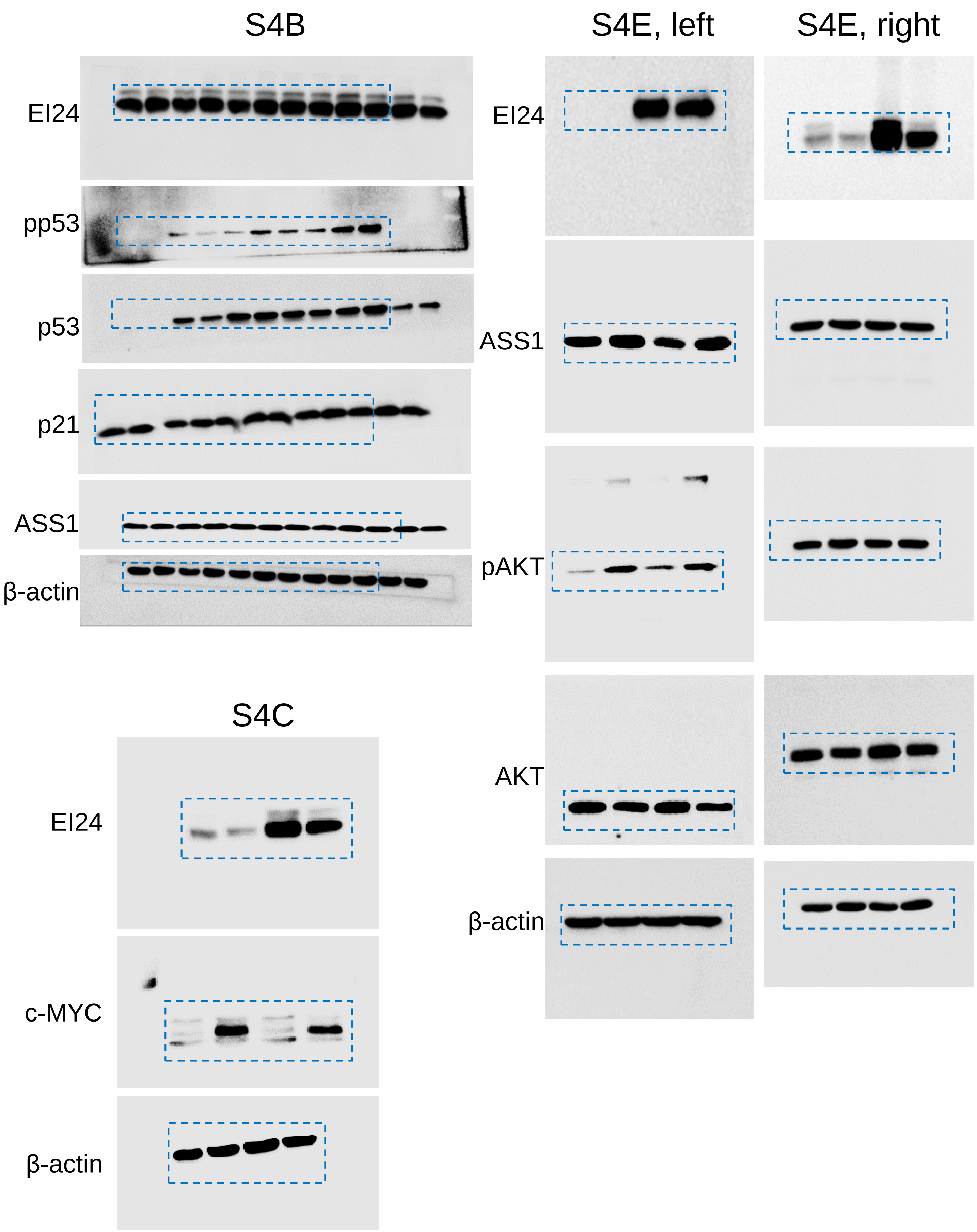
S4B
EI24
pp53
p53
p21
ASS1
β-actin
S4C
EI24
c-MYC
β-actin
S4E, left
S4E, right
EI24
ASS1
pAKT
AKT
β-actin
